# Supplementary material for: Late Pleistocene human genome suggests a local origin for the first farmers of central Anatolia
Source: Nat Commun. 2019 Mar 19;10:1218. doi: 10.1038/s41467-019-09209-7 (PMC6425003; doi:10.1038/s41467-019-09209-7)
Supplement: Supplementary file 1 — Supplementary Information [file 41467_2019_9209_MOESM1_ESM.pdf]

Supplementary Information

Late Pleistocene human genome suggests a local origin for the first farmers of central Anatolia

Feldman et al.

Supplementary Figures

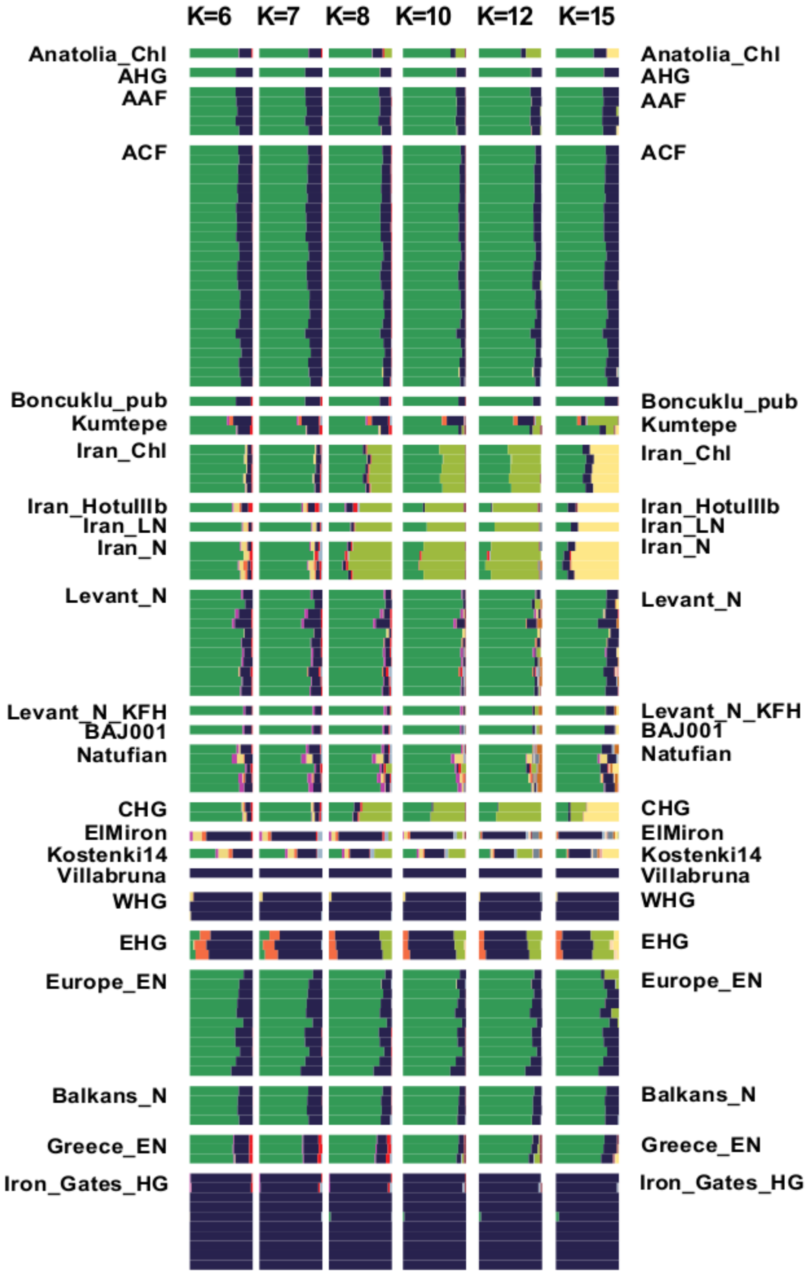

Supplementary Figure 1. ADMIXTURE analysis. ADMIXTURE analysis (Materials and Methods) computed from 2,706 present-day and 594 ancient individuals is shown (K = 6-8; 10; 12; 15). A selected set of ancient individuals are plotted. For ADMIXTURE plot of all individuals (K=10) see Supplementary Figure 9. AHG, AAF and ACF compose of similar main ancestry components, maximized in Natufians (green) and WHG (blue). Source data are provided as a Source Data file.

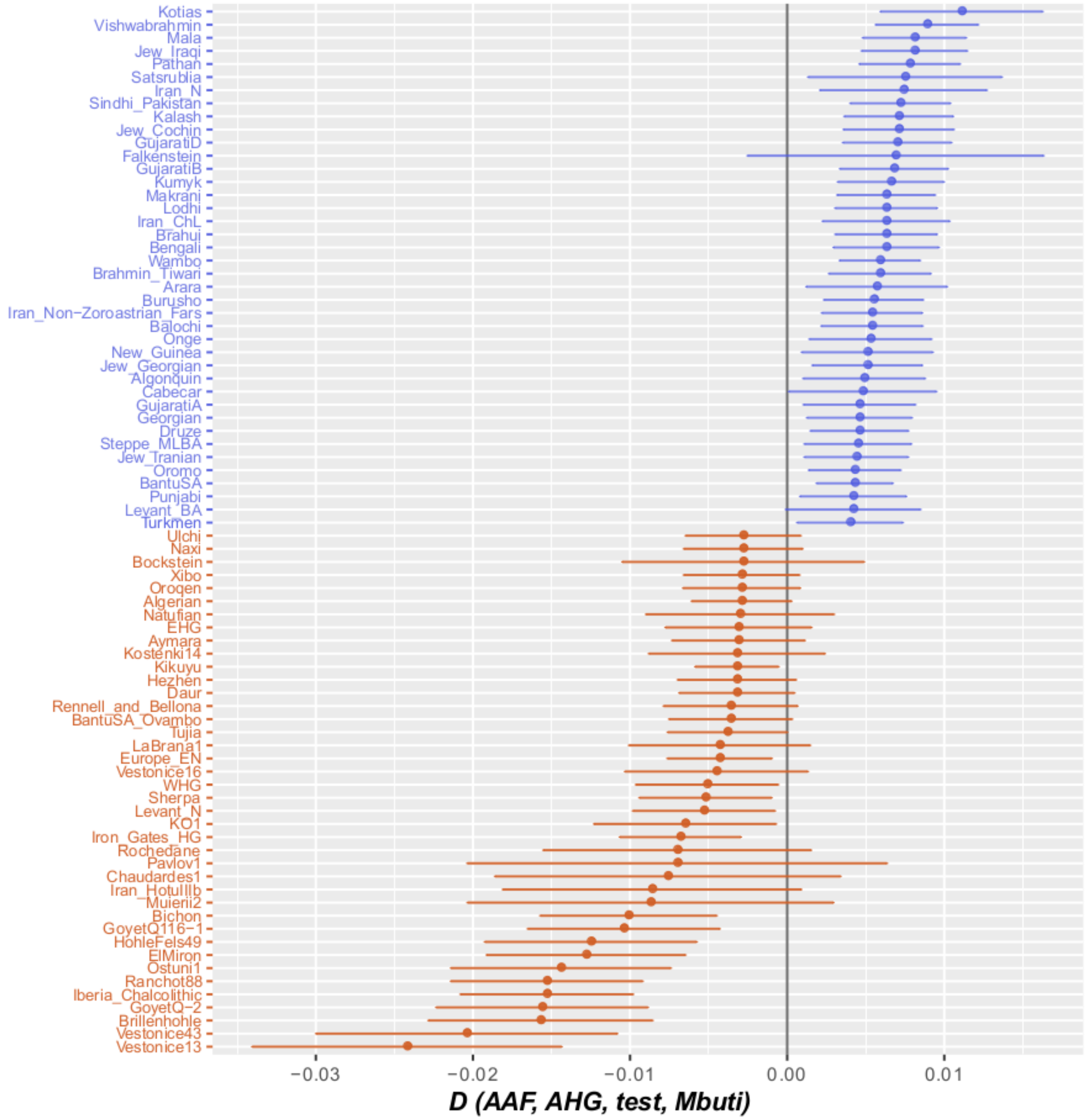

Supplementary Figure 2. AAF have excess allele sharing with Iranian/Caucasus related populations compared with AHG. We plot the populations with the 40 most positive (blue) and 40 most negative values (orange) of *D(AAF, AHG; test, Mbuti)* with ±1 standard errors estimated by 5 cM block jackknifing (represented by the horizontal bars). Positive values indicate that "test" shares more alleles with *AAF* than with *AHG*, and negative values that it shares more with *AHG* than *AAF* (analysis was restricted to individuals > 30,000 SNPs).

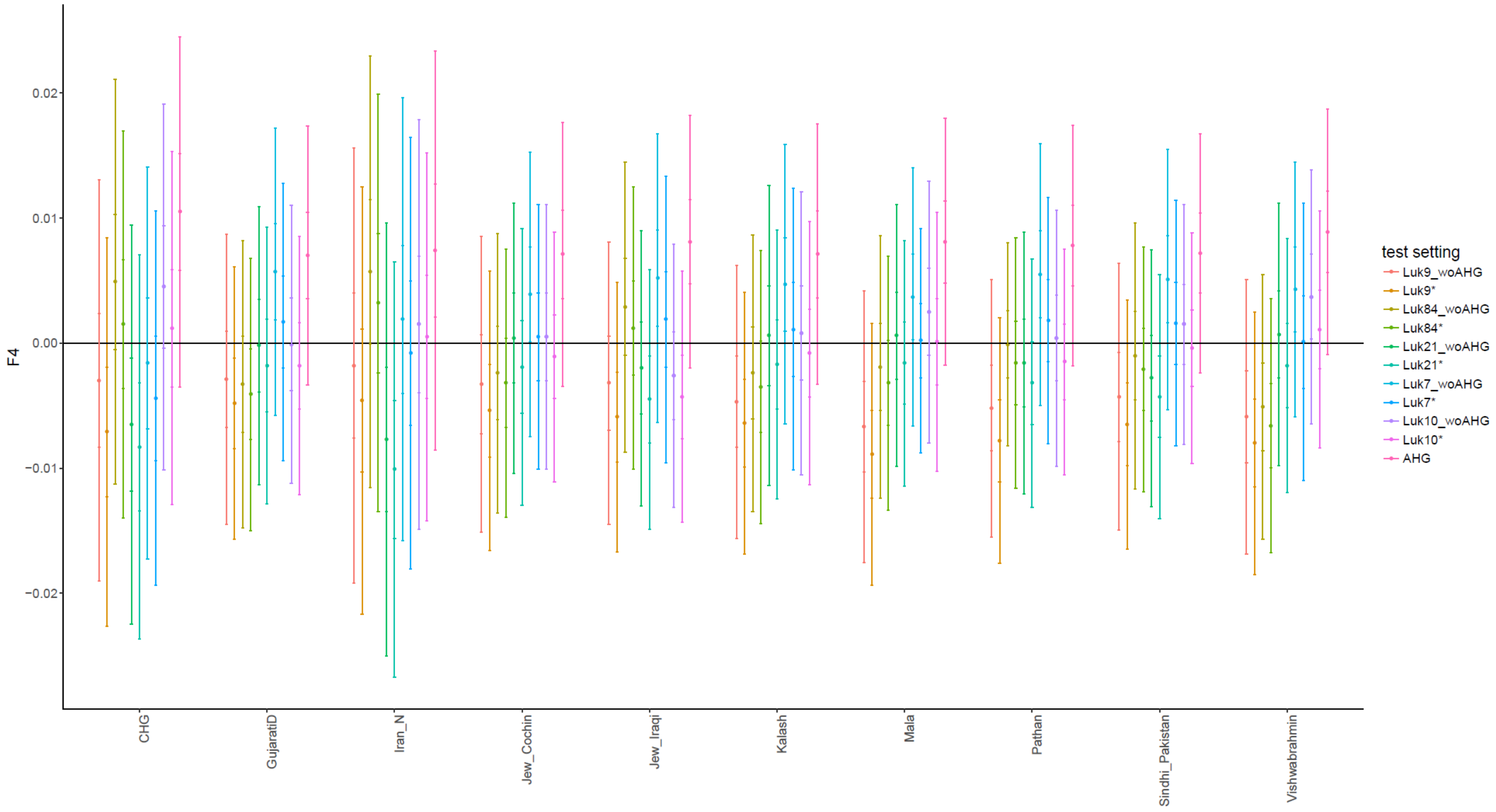

Supplementary Figure 3. Permutation test of the D statistic of the form *D(AAF*, AHG*; test, Mbuti)*. We plot the populations with the 10 most positive values of *D(AAF, AHG; test, Mbuti)* and the permutation values for the same populations with ±1 and ±3 standard errors estimated by 5 cM block jackknifing (represented by the inner and outer vertical bars respectively). In each test setting "*AAF**" either includes AHG and all AAF individuals except the individual marked with an asterisk or all AAF individuals (and not AHG) except the individual marked with "_woAHG". The test with the original population labels is indicated in pink (analysis was restricted to individuals > 30,000 SNPs). All permutated settings are consistent with a negative *D*-score or 0 within ±1 standard errors while the original *D*-scores for these populations resulted in a positive *D*-score within ±1 standard errors.

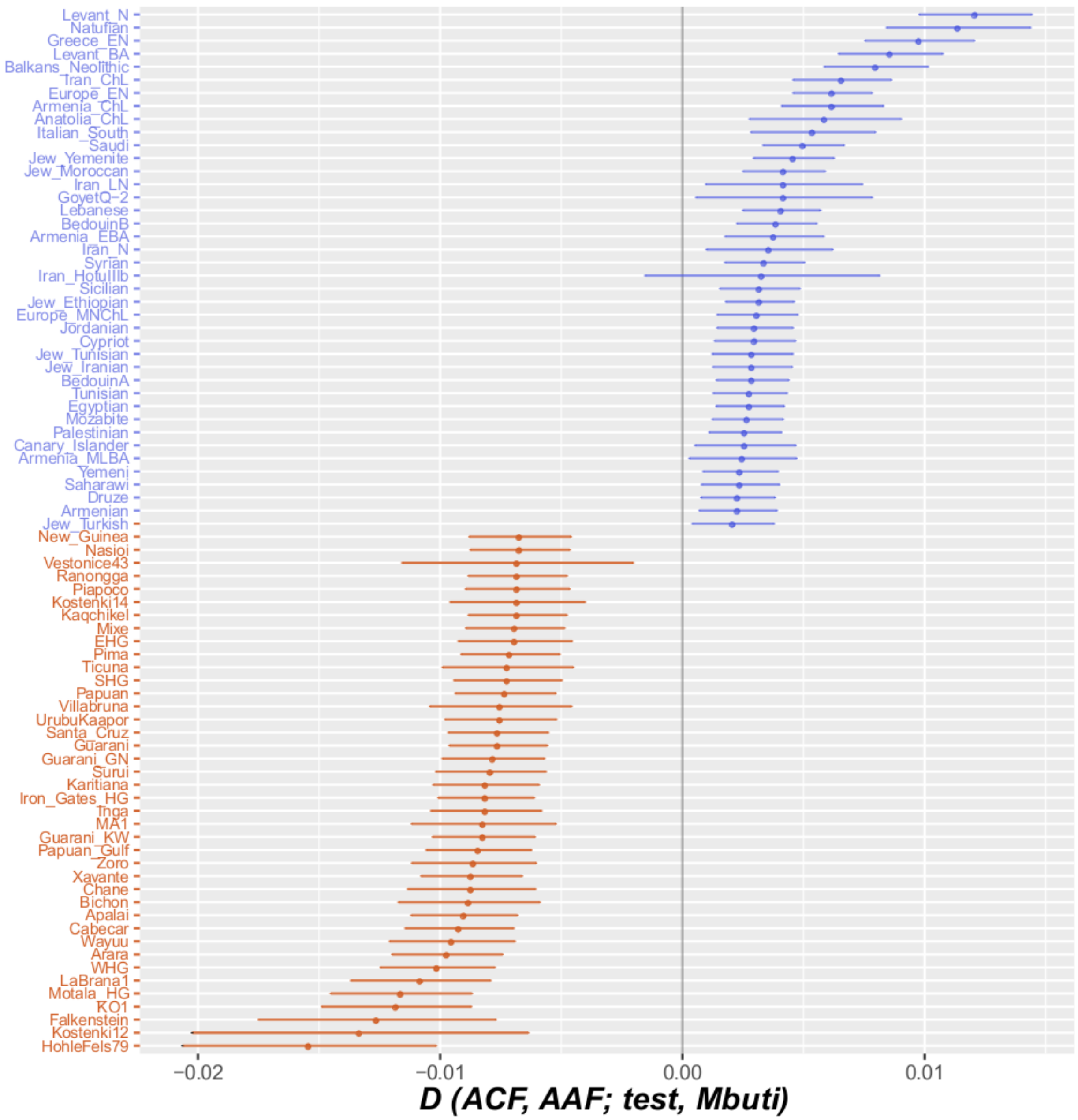

Supplementary Figure 4. ACF have excess allele sharing with Levantine related populations compared with AAF. We plot the populations with the 40 most positive (blue) and 40 most negative values (orange) of *D(ACF, AAF; test, Mbuti)* with ±1 standard errors estimated by 5 cM block jackknifing (represented by the horizontal bars). Positive values indicate that “test” shares more alleles with *ACF* than with *AAF*, and negative values that it shares more with *AAF* than *ACF* (analysis was restricted to individuals > 30,000 SNPs).

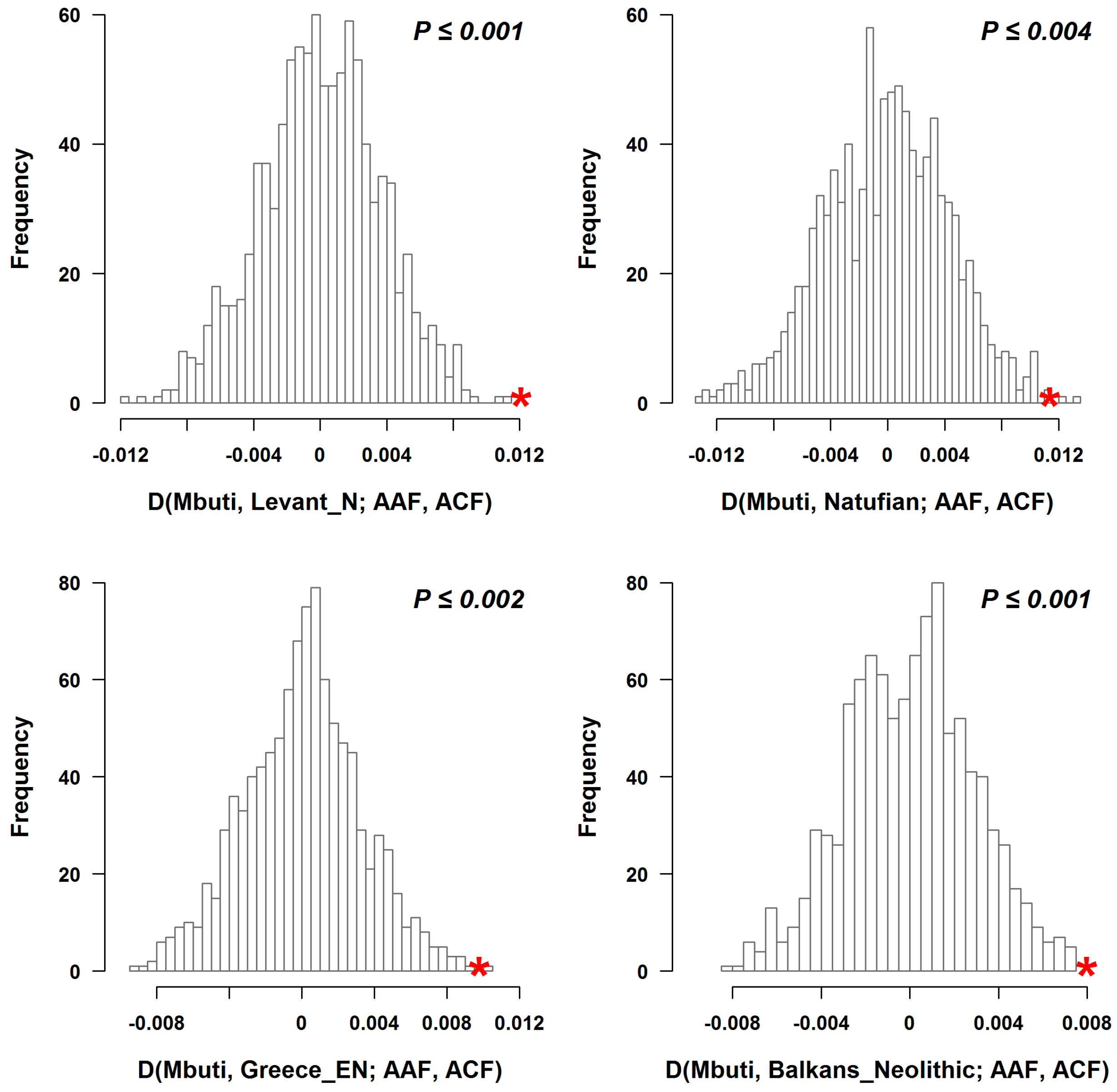

Supplementary Figure 5. Permutation test of the *D-statistic* of the form *D(ACF*, AAF*; test, Mbuti)*. We plot the empirical null distribution of *D-statistics* based on 1000 permutation tests performed for each of the four "test" populations that had the most positive values in the original observed statistic (*Levant_N*, *Natufian*, *Greece_EN*, *Balkans_Neolithic*). In each test individuals were randomly shuffled between the *ACF** and *AAF** groups. The *D-statistic* with the original population labels is marked with an asterisk. Empirical *P*-values are indicated for each plot and were calculated by dividing the number of permutations that resulted in a value equal to or greater than the original observation by the total number of permutations. In all cases, the observed value is at the top < 1% tail.

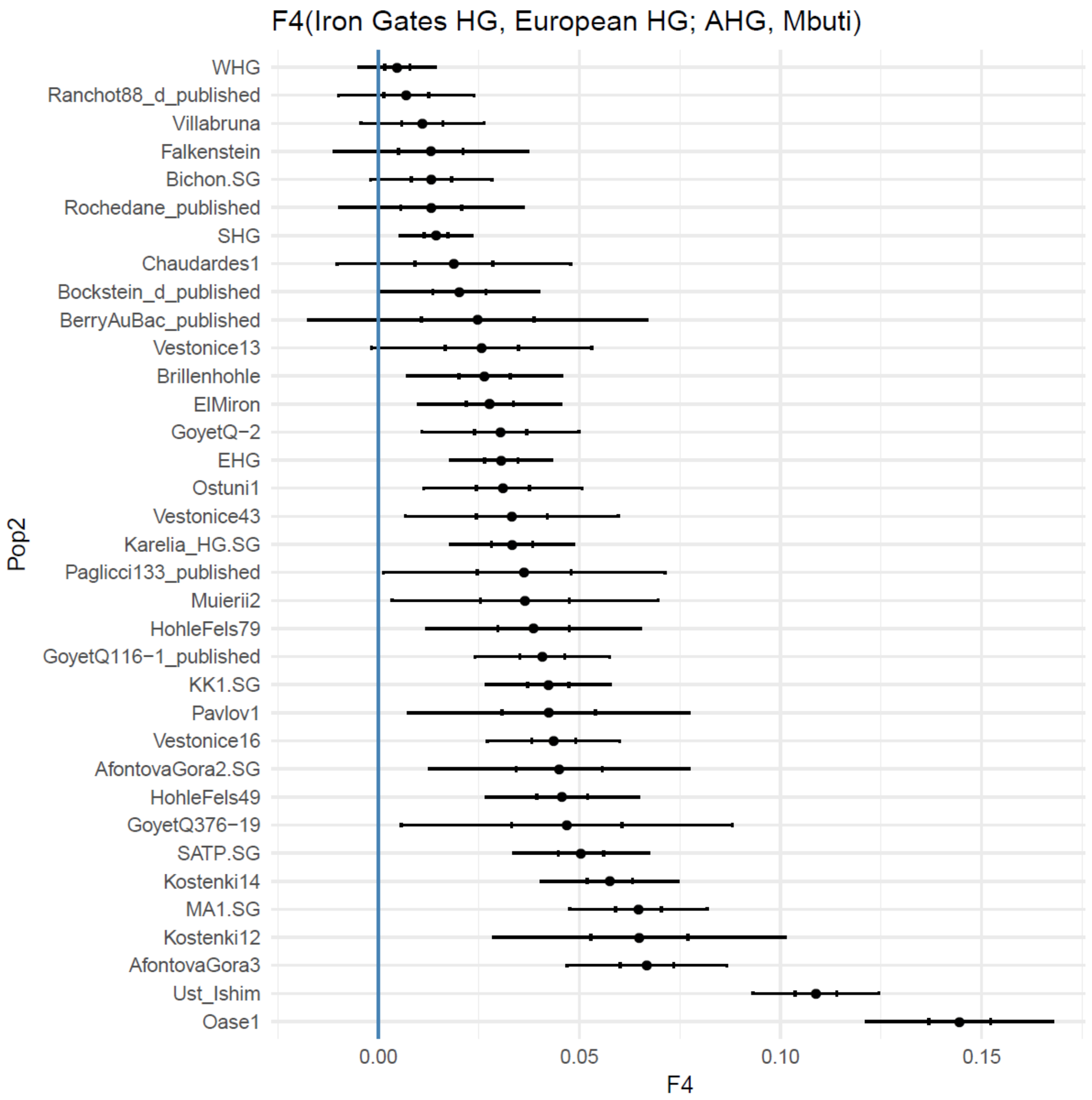

Supplementary Figure 6. Iron Gates HG show higher genetic affinity to AHG than all the other European HG. We plot the *f4* values of *D(Iron Gates HG, European HG; AHG, Mbuti)* with ±1 and ±3 standard errors estimated by 5 cM block jackknifing (represented by the inner and outer horizontal bars respectively). Positive values indicate that "*AHG*" shares more alleles with *Iron Gates HG* than with the tested *European HG* (analysis was restricted to individuals > 30,000 SNPs).

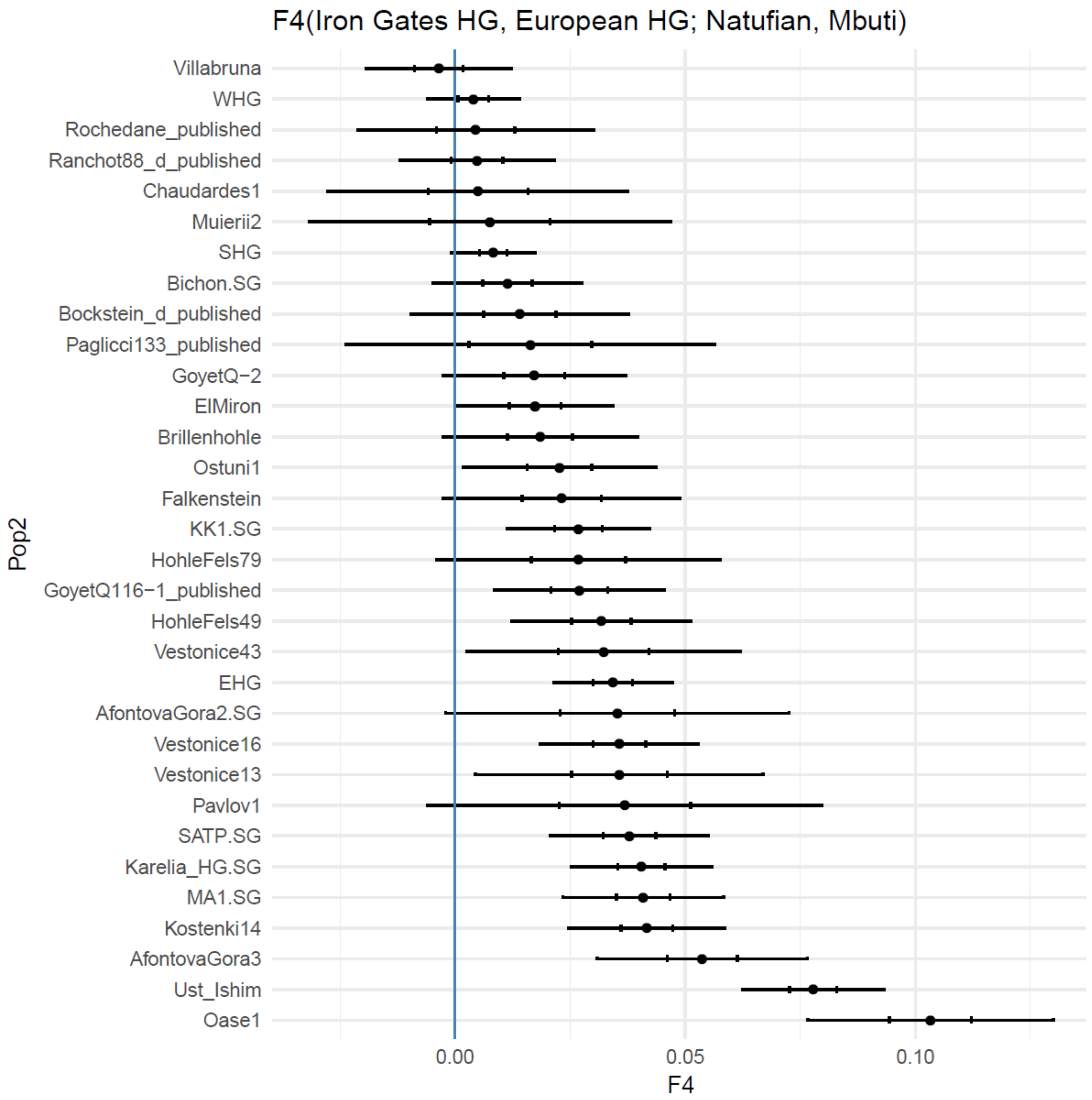

Supplementary Figure 7. Iron Gates HG show a higher genetic affinity to Natufians than all the other European HG but Villabruna. We plot the *f4* values of *D(Iron Gates HG, European HG; Natufian, Mbuti)* with ±1 and ±3 standard errors estimated by 5 cM block jackknifing (represented by the inner and outer horizontal bars respectively). Positive values indicate that "*Natufian*" shares more alleles with *Iron Gates HG* than with the tested *European HG* (analysis was restricted to individuals > 30,000 SNPs).

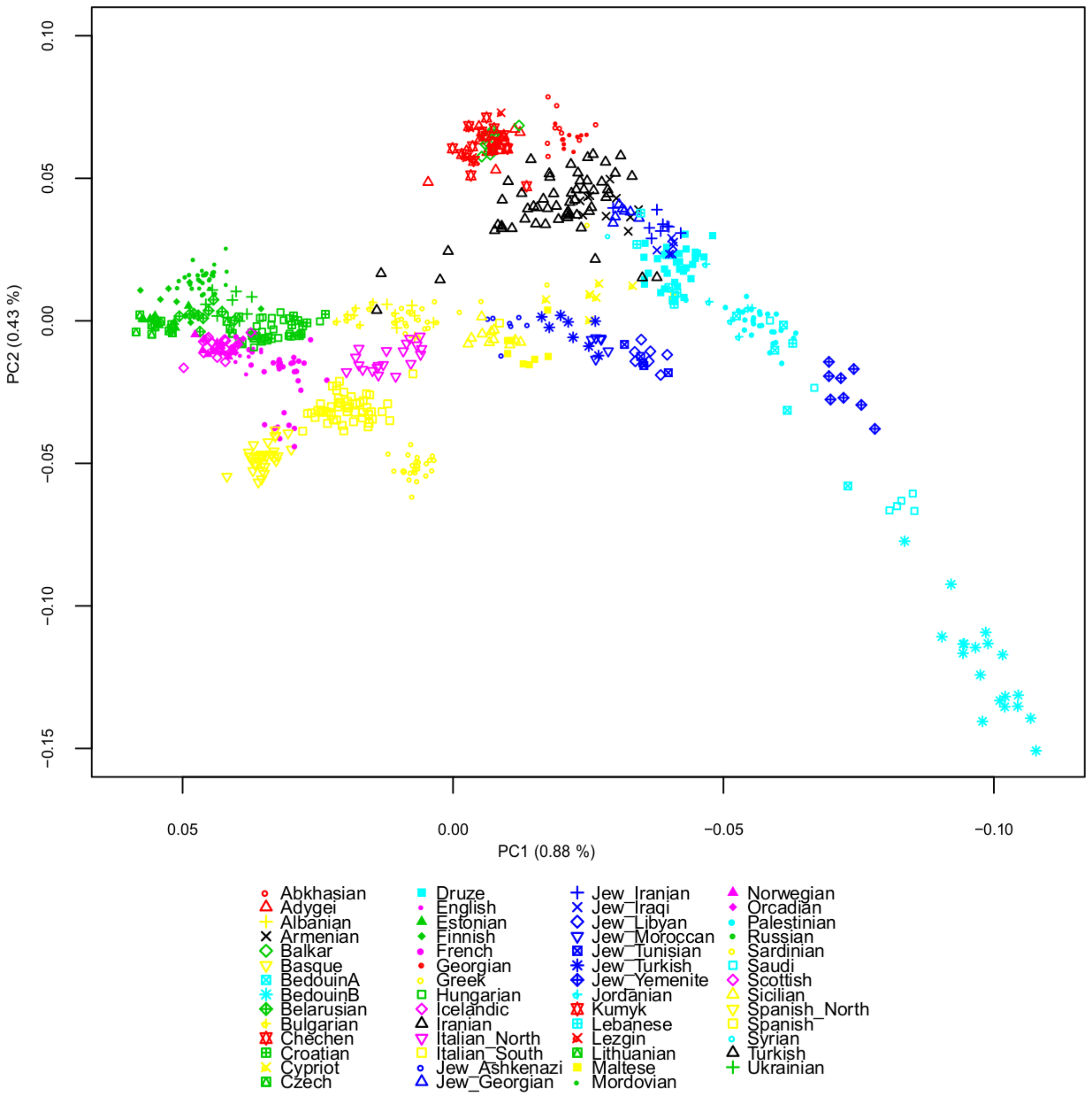

Supplementary Figure 8. PCA plot of present-day west Eurasian populations. The two first principal components computed for 67 published present day west Eurasian populations are shown. An estimated 0.88 % of the variation is explained by the first principal component (PC1) and 0.43 % is explained by the second principal component (PC2). Source data are provided as a Source Data file.

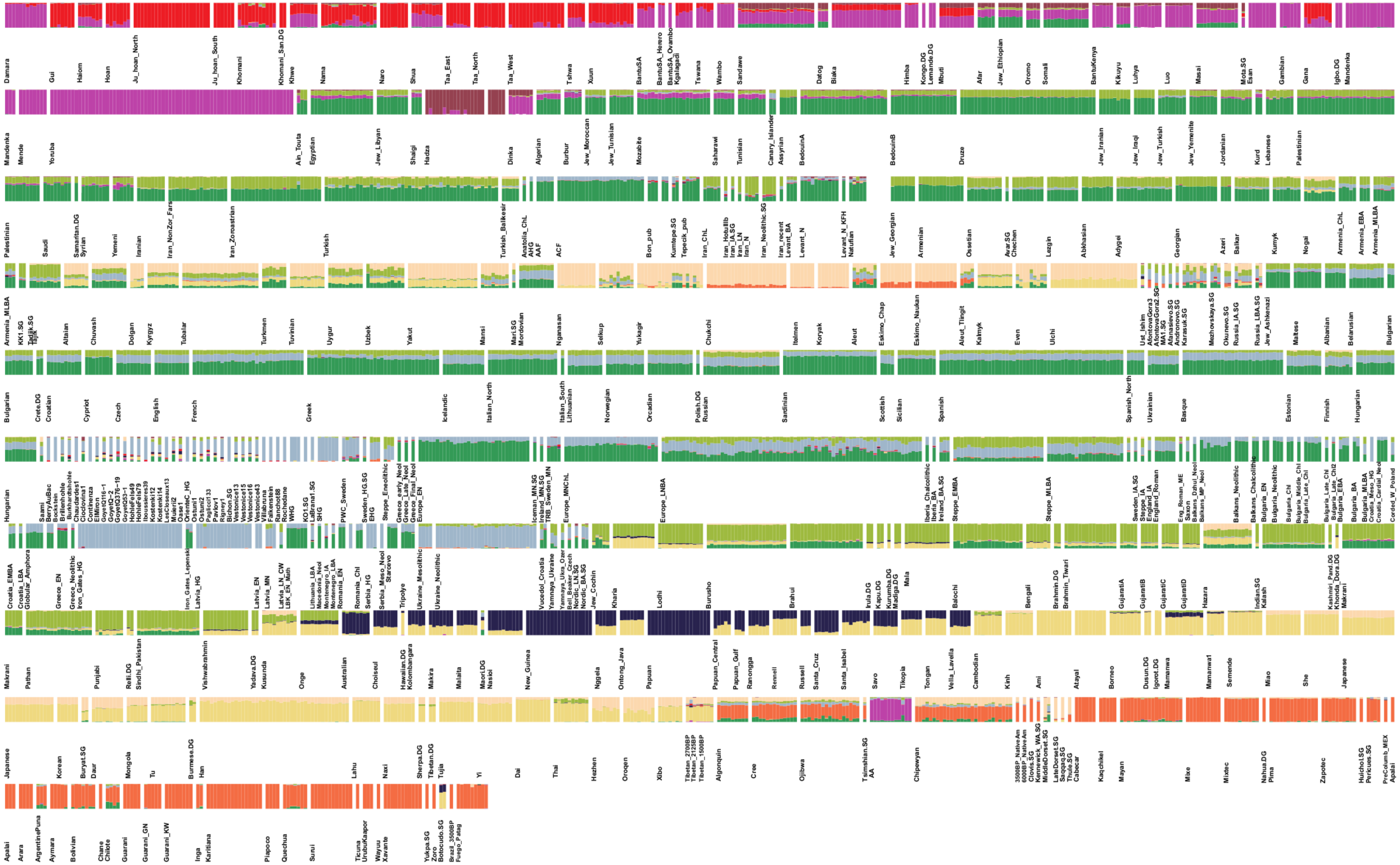

Supplementary Figure 9. ADMIXTURE plot of all individuals analyzed. ADMIXTURE analysis (Methods) computed from 2,706 present-day and 594 ancient individuals is shown (K = 10). Source data are provided as a Source Data file.

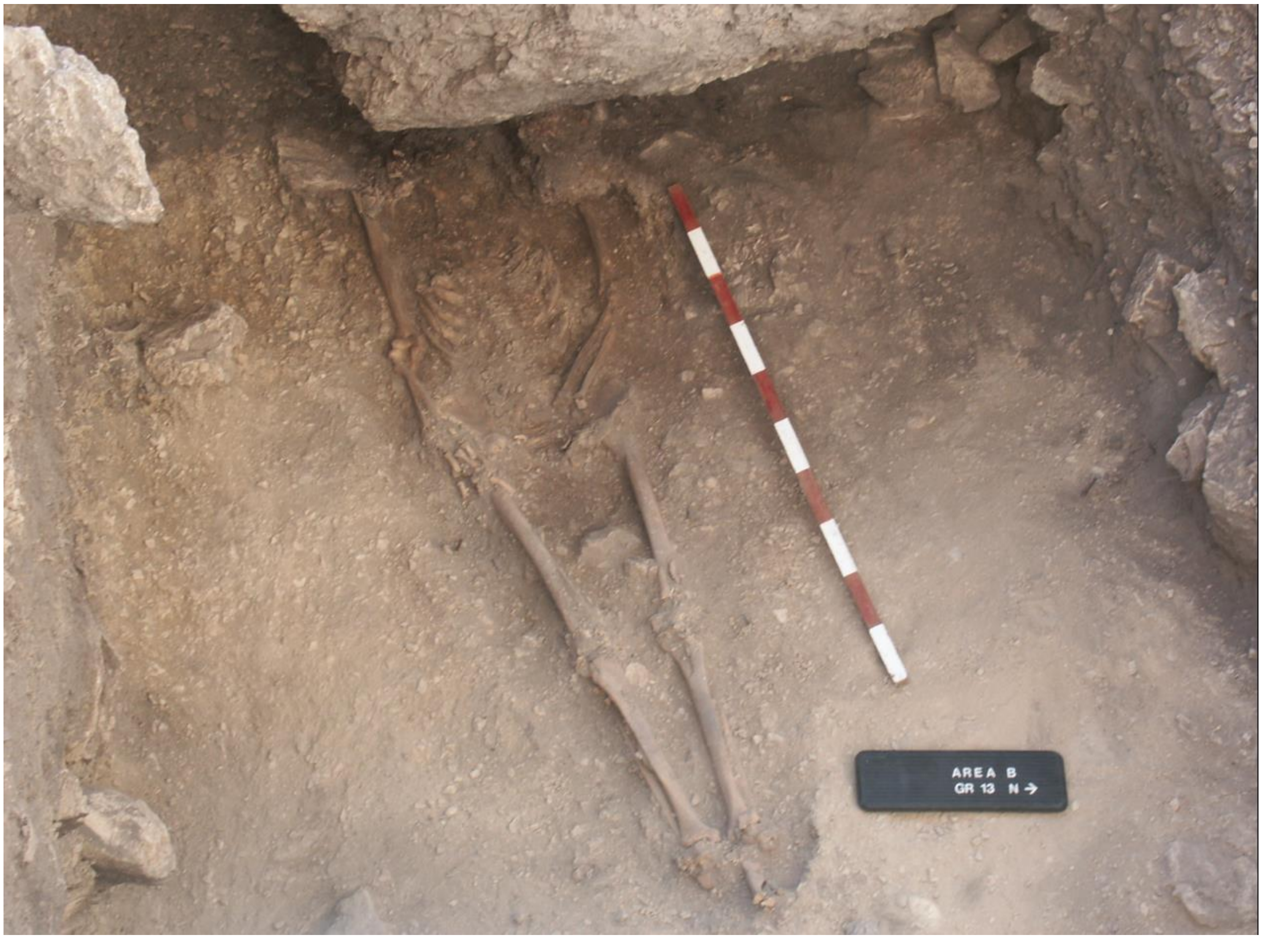

Supplementary Figure 10. View of the sampled Anatolian Epipaleolithic hunter-gatherer (AHG/ZBC). Excavated from grave 13 in Pınarbaşı. Photo by Douglas Baird.

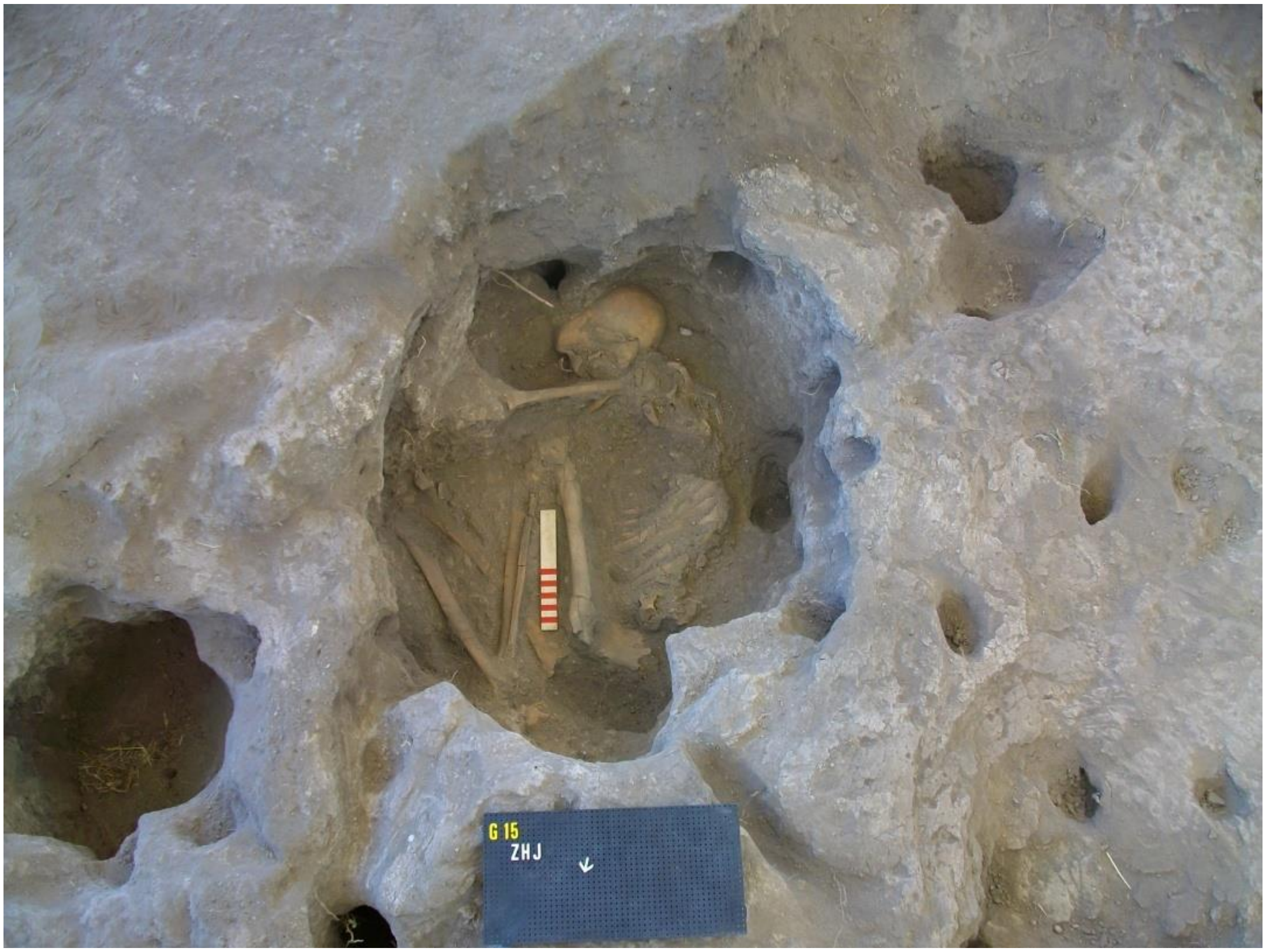

Supplementary Figure 11. View of sampled AAF individual ZHJ. Excavated from grave 15, Boncuklu[11]. Photo by Douglas Baird/Boncuklu project.

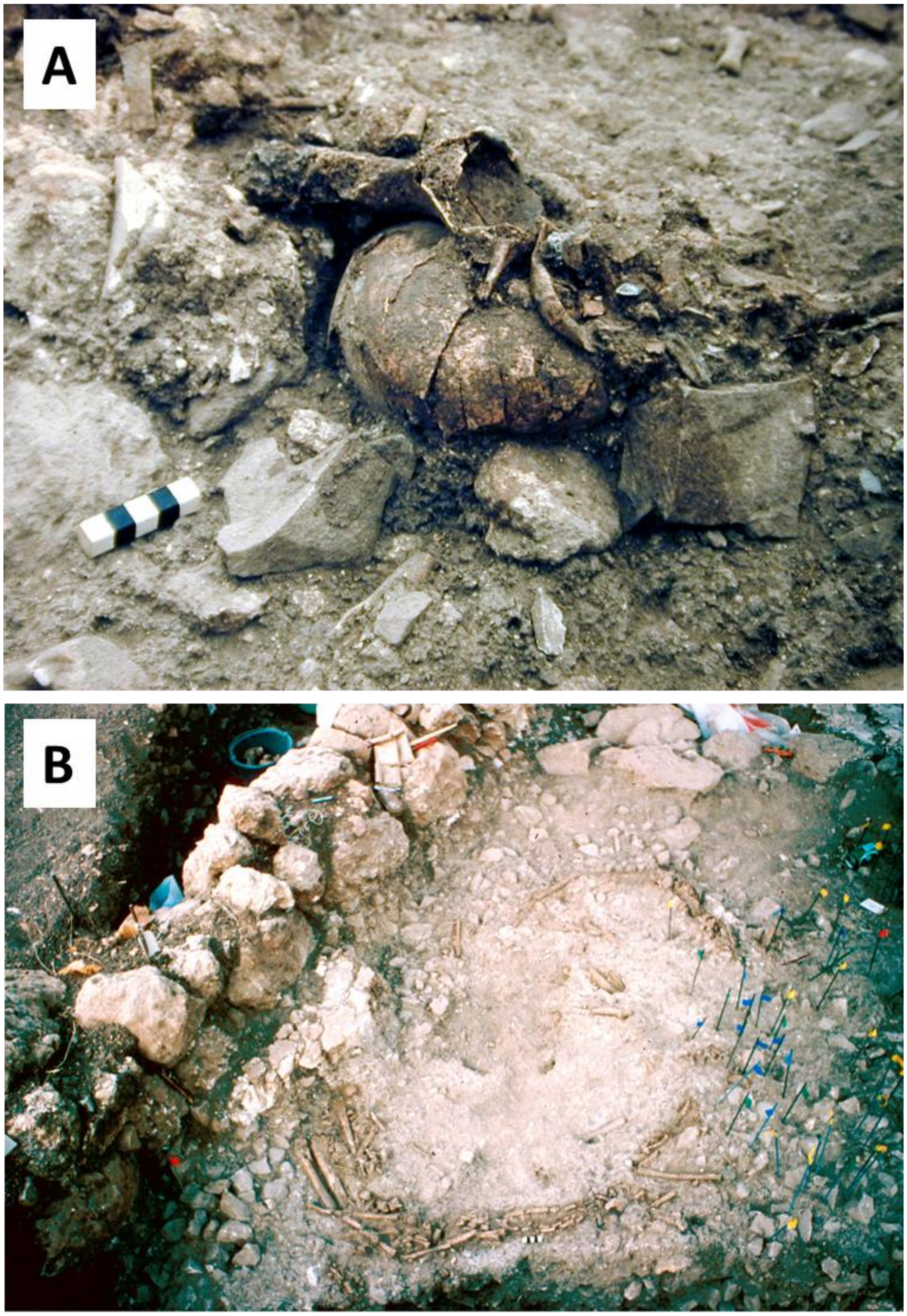

Supplementary Figure 12. Kfar HaHoresh site and sampled early farmer KFH2 (A) View of sampled infant skull KFH2 *in situ* in L1003 of Kfar HaHoresh. Scale 5cm. **(B)** View of upper levels of multiple grave in L1003 (underlying L1001), showing intentional arrangement of human bones. Scale 5cm.

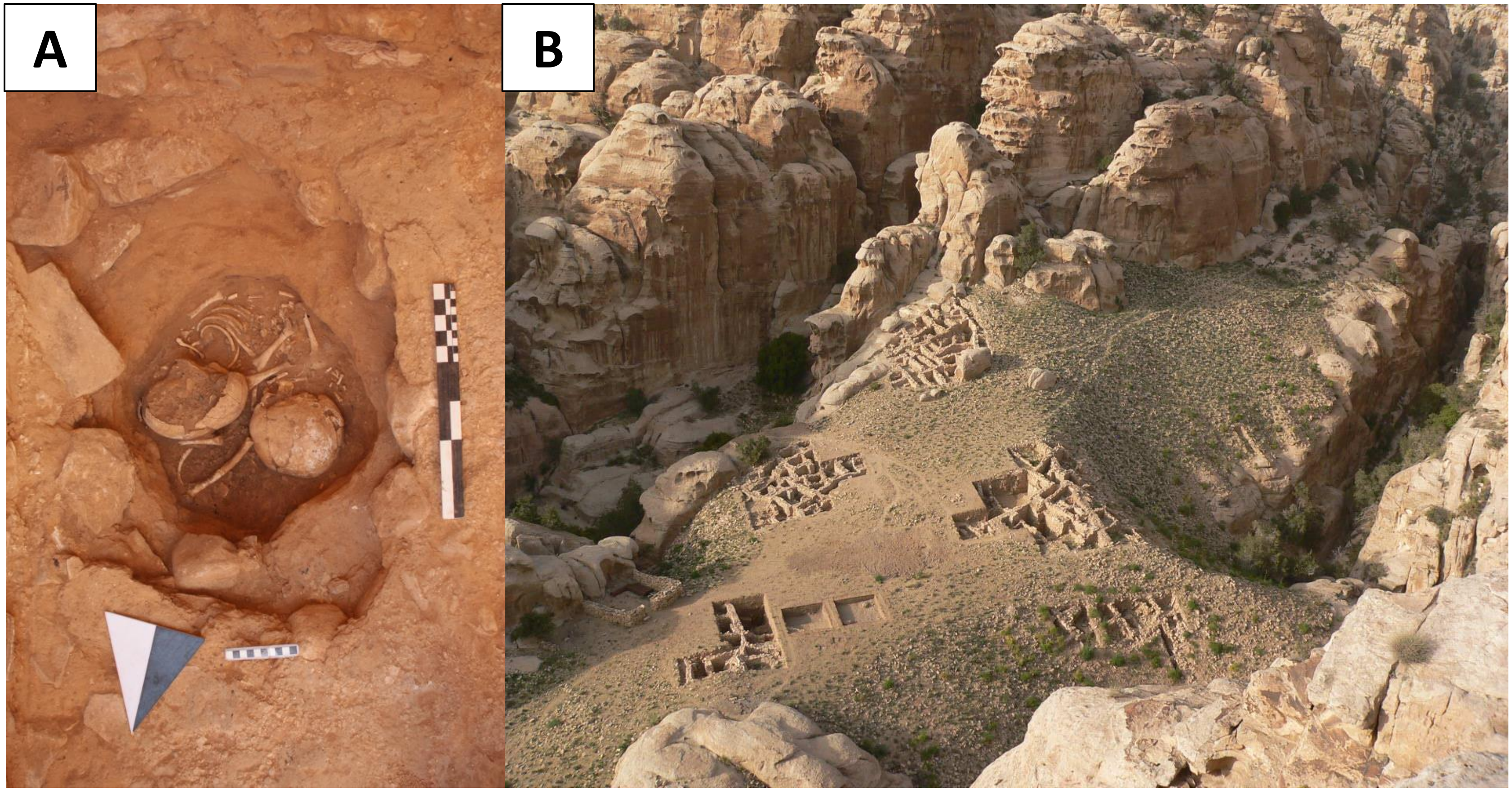

Supplementary Figure 13. The site of Ba'ja and the double infant burial. (A) View of the double infant burial (Loc. 405 of Room 35; individuals BAJ001 and BAJ002); Photo by Benz. **(B)** View on the Late Pre-Pottery Neolithic B site of Ba'ja (southern Jordan) from the top of the mountains. Ba'ja Neolithic Project, Photo by Borowski.

5

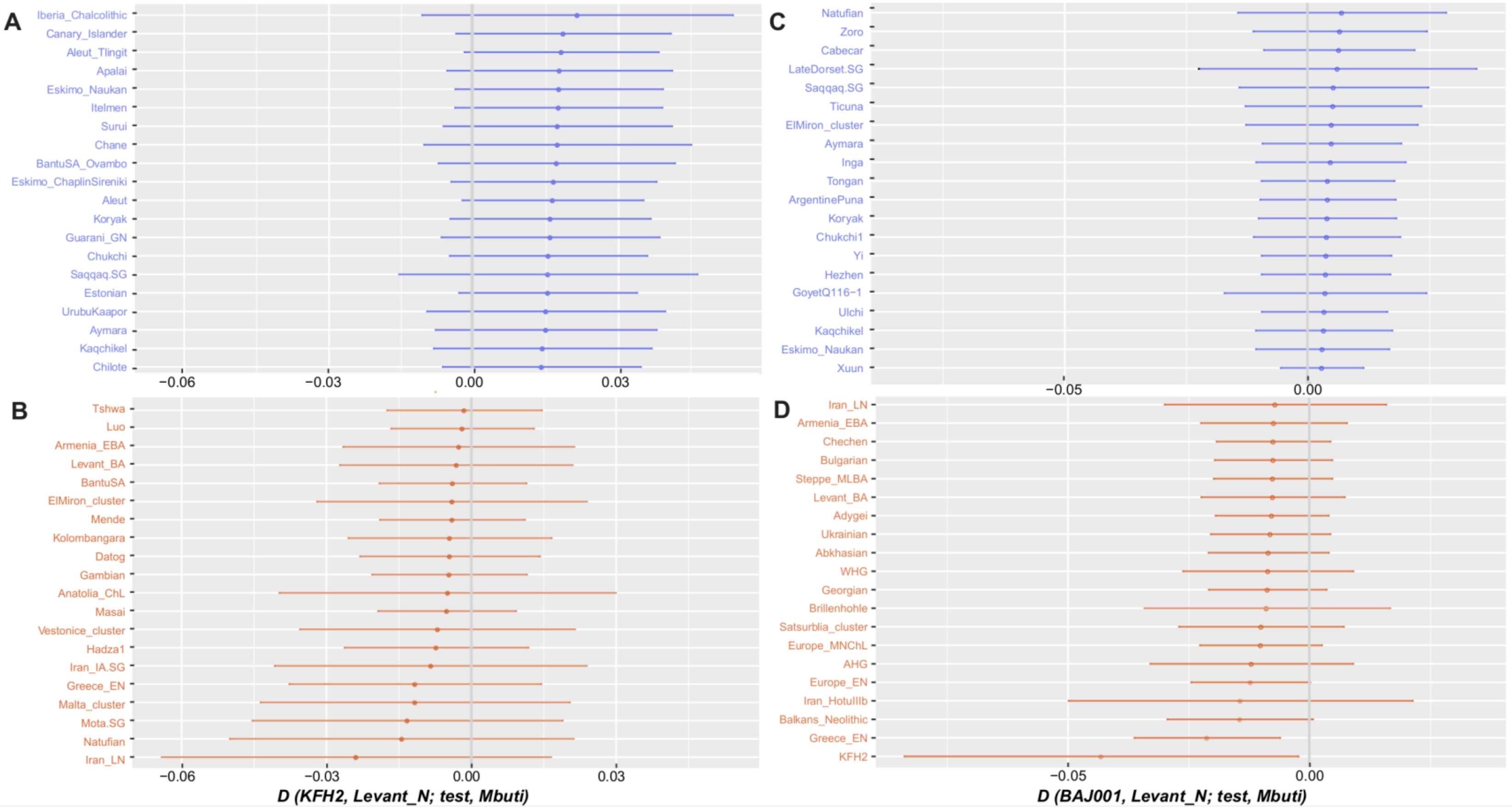

Supplementary Figure 14. Symmetry testing of the newly reported KFH2 and BAJ001 individuals with previously reported Levantine Neolithic individuals (grouped and labeled Levant_N). We plot the 20 most positive (blue) and the 20 most negative values (orange) of the *D*-statistic of the form *D(KFH2/BAJ001, Levant_N; test, mbuti)* with ±3 standard errors (represented by the horizontal lines) estimated by 5 cM block jackknifing. "*test*" populations include versatile global ancient and modern populations. Positive values indicate that "*test*" shares more alleles with *KFH2/BAJ001* than with *Levant_N*, and negative values that it shares more with *Levant_N* than *KFH2/BAJ001*. Results for all tested quadruples can be found in Supplementary Data 4 (analysis was restricted to individuals > 30,000 SNPs). (**A**) The 20 most positive values of the *D* statistic of the form *D(KFH2, Levant_N; test, mbuti)* are plotted. (**B**) The 20 most negative values of the *D* statistic of the form *D(KFH2, Levant_N; test, mbuti)* are plotted. (**C**) The 20 most positive values of the *D* statistic of the form *D(BAJ001, Levant_N; test, mbuti)* are plotted. (**D**) The 20 most negative values of the *D* statistic of the form *D(BAJ001, Levant_N; test, mbuti)* are plotted.

Supplementary Tables

Supplementary Table 1. ^{14}C radiocarbon dating performed for this study. Carbon dated at the CEZ Archaeometry gGmbH, Mannheim, Germany. The ^{14}C ages are given in BP (before present; meaning years before 1950). The calibrated dates are shown in columns “Cal 1-sigma” and “Cal 2-sigma” using the 1-sigma and 2-sigma uncertainty of the ^{14}C ages, respectively. The d^{13}C value was obtained from the isotope determination in the AMS system with a typical uncertainty of 2%. This value may be influenced by isotope fractionation in the ion source and during graphitization and is only used for fractionation correction. Hence, this value is not comparable to the one obtained in a stable isotope IRMS and should not be used for further data interpretation.

Individual	Experiment number (MAMS)	C14 date (BP)	SE	Sigma 13C AMS [‰]	Cal 1-sigma (BCE)	Cal 2-sigma (BCE)	C:N	C (%)	Collagen (%)	Tissue
**KFH2**	31616	8,638	24	-9.1	7,647-7,594	7,712-7,589	3.3	40.2	1.5	Petrous bone
**ZBC**	30693	12,890	40	-16.7	13,530-13,335	13,647-13,284	3.3	37.7	0.8	Phalanx bone

Supplementary Table 2. Nuclear and mitochondrial contamination estimates. For each individual newly reported and analyzed in this study mitochondrial contamination estimates calculated with schmutzi (Materials and Methods) are given. For genetic males the nuclear contamination estimate is provided (Materials and Methods). The levels of DNA damage are given as the deamination level at the 5' terminal position of the mapped reads.

**Individual ID**	**Site**	**Nuclear contamination estimate**	**SE**	**Mitochondrial contamination estimate**	**Deamination at 5' terminal position (%)**
ZBC	Pınarbaşı	0.005	0.002	0.01 (0.00 -0.02)	11
KFH2	Kfar HaHoresh	NA	NA	0.06 (0.04-0.08)	26
BAJ001	Ba'ja	NA	NA	0.01(0.00-0.02)	28
ZHAG	Boncuklu	NA	NA	0.01 (0.00-0.02)	11
ZMOJ	Boncuklu	0.009	0.006	0.03 (0.02-0.04)	22
ZKO	Boncuklu	0.022	0.007	0.01 (0.00-0.02)	16
ZHJ	Boncuklu	NA	NA	0.01 (0.00-0.02)	9
ZHAJ	Boncuklu	NA	NA	0.03 (0.02-0.04)	16

Supplementary Table 3. Test of cladeness between AHG and late Pleistocene/early Holocene populations. The *D*-statistics of the form *D* (*AHG, pop1; pop2, Mbuti*) is shown where “*pop1*” and “*pop2*” are late Pleistocene/early Holocene groups from Europe or the Near East. Positive *D*-values (Z>3) indicate that “*pop2*” shares more alleles with AHG compared to “*pop1*” and negative *D*-values (Z<-3) indicate that “*pop2*” shares more alleles with “*pop1*” compared to AHG. The *Z* scores were calculated from a 5 cM block jackknifing standard error. The number of SNP positions covered in all four tested populations is given in column ‘nSNPs’.

**Pop1**	**Pop2**	***D***	***Z***	**nSNPs**
WHG	Levant_N	0.0458	9.953	384,901
WHG	Natufian	0.0285	4.822	236,731
WHG	Iran_N	0.0087	1.613	390,457
WHG	EHG	-0.0693	-14.094	453,196
Levant_N	WHG	0.0439	9.038	384,901
Levant_N	Natufian	-0.0365	-5.56	215,370
Levant_N	Iran_N	0.0103	1.779	341,358
Levant_N	EHG	0.0448	8.965	374,568
Natufian	WHG	0.0545	9.585	236,731
Natufian	Levant_N	-0.0084	-1.364	215,370
Natufian	Iran_N	0.0331	4.766	214,816
Natufian	EHG	0.057	8.804	230,917
Iran_N	WHG	0.075	13.793	390,457
Iran_N	Levant_N	0.0784	14.371	341,358
Iran_N	Natufian	0.0725	10.499	214,816
Iran_N	EHG	0.0394	6.598	379,907
EHG	WHG	-0.043	-8.546	453,196
EHG	Levant_N	0.0691	14.148	374,568
EHG	Natufian	0.0568	8.846	230,917
EHG	Iran_N	-0.0041	-0.692	379,907

Supplementary Table 4. Summary of best fitting qpAdm admixture models of key ancient populations. For each target population the proportions estimated by the best fitting admixture models (Pval > 0.05) are given with their standard errors estimated by 5 cM block jackknifing. **‘**Ref’ 1-3 indicate the ancestral sources (reference) used to model the target populations. The abbreviations of the population names are listed in Supplementary Data 2. We define the ‘Basic set’ of outgroups as: *Han; Onge; Mbuti; Natufian; Kostenki14; Mala; Mixe*. When *Natufian* was used as a source population, the present-day *BedouinB* Near-Eastern population was used as an outgroup in the ‘Basic set’ instead.

**Target**	**Ancestral sources**			**Mixture proportions (%)**			**Standard errors (%)**				
	**Ref1**	**Ref2**	**Ref3**	**Ref1**	**Ref2**	**Ref3**	**Ref1**	**Ref2**	**Ref3**	**Pval (rank - 1)**	**Outgroups**
AHG	Levant_N	WHG		47.9	52.1		4.5	4.5		0.158	Basic set
AHG	Levant_N	WHG	Iran_N	46.4	41.7	11.9	4.6	7.1	6.9	0.296	Basic set
AAF	AHG	Iran_N		89.7	10.3		3.9	3.9		0.296	Basic set; WHG; EHG
ACF	AAF	Levant_N		78.7	21.3		3.5	3.5		0.606	Basic set
Iron_Gates_HG	AHG	WHG	EHG	25.8	62.9	11.3	5.0	7.4	3.3	0.308	Basic set
Iron_Gates_HG	Natufian	WHG	EHG	11.1	78.0	10.9	2.2	4.6	3.0	0.589	Basic set
Levant_Neol	AAF	Natufian		17.8	82.2		6.4	6.4		0.288	Basic set; WHG; EHG
Levant_Neol	AHG	Natufian		16.3	83.7		6.7	6.7		0.074	Basic set; WHG; EHG

Supplementary Table 5. qpADM Admixture models of the Anatolian hunter-gatherer (AHG). The proportions estimated for each ancestral source (Ref1-4) used to model the target population AHG are given. Fitting models (Pval > 0.05; and admixture proportions are feasible) are highlighted in green. When resolution is lacking to determine whether a mixture proportion is required the source population, proportion and Standard error are highlighted in lighter green. The fitting models with minimal waves of ancestry are marked in bold. The standard errors were estimated by 5 cM block jackknifing. The abbreviations of the population names are listed in Supplementary Data 2. The ‘Basic set’ of outgroup populations (*Han; Onge; Mbuti; Natufian; Kostenki14; Mala; Mixe)* was used.

**Ancestral sources**				**Mixture proportions (%)**				**Standard errors (%)**				
**Ref1**	**Ref2**	**Ref3**	**Ref4**	**Ref1**	**Ref2**	**Ref3**	**Ref4**	**Ref1**	**Ref2**	**Ref3**	**Ref4**	**Pval (rank–1)**
**Levant_N**	**WHG**			**47.9**	**52.1**			**4.5**	**4.5**			**1.58E-01**
Levant_N	EHG			68.7	31.3			3.1	3.1			1.15E-05
Levant_N	Iran_N			55.7	44.3			5.1	5.1			3.31E-07
Iran_N	WHG			27.1	72.9			15.9	15.9			4.59E-21
Iran_N	EHG			110.0	-10.0			8.7	8.7			2.27E-29
Levant_N	WHG	EHG		48.2	49.5	2.4		5.2	10.4	6.5		1.31E-01
Levant_N	WHG	Iran_N		46.4	41.7	11.9		4.6	7.1	6.9		2.96E-01
Levant_N	EHG	Iran_N		59.0	21.3	19.7		4.8	4.7	7.4		1.16E-03
Iran_N	WHG	EHG		28.0	110.7	-38.7		11.4	14.6	7.5		8.95E-12
Levant_N	WHG	Iran_N	EHG	46.7	39.7	11.5	**2.2**	5.4	10.7	7.0	6.6	2.35E-01

Supplementary Table 6. qpADM Admixture models of the Anatolian Aceramic farmers (AAF). The proportions estimated for each ancestral source (Ref1-3) used to model the target population AAF are given. Fitting models (Pval > 0.05; and admixture proportions are feasible) are highlighted in green and in bold letters. The standard errors were estimated by 5 cM block jackknifing. The abbreviations of the population names are listed in Supplementary Data 2. We define the 'Basic set' of outgroup populations as *Han; Onge; Mbuti; Natufian; Kostenki14; Mala; Mixe*.

**Ancestral sources**			**Mixture proportions (%)**			**Standard errors (%)**				
**Ref1**	**Ref2**	**Ref3**	**Ref1**	**Ref2**	**Ref3**	**Ref1**	**Ref2**	**Ref3**	**Pval (rank – 1)**	**Outgroups**
**AHG**	**Iran_N**		**91.4**	**8.6**		**7.2**	**7.2**		**5.38E-02**	**Basic set**
**AHG**	**Iran_N**		**89.7**	**10.3**		**3.9**	**3.9**		**2.96E-01**	**Basic set; Levant_N;WHG;EHG**
AHG	EHG		98.7	1.3		4.0	4.0		1.37E-02	Basic set
AHG	Levant_N		108.1	-8.1		9.8	9.8		5.76E-02	Basic set
AHG	WHG		103.7	-3.7		9.9	9.9		1.07E-02	Basic set
WHG	EHG		144.4	-44.4		8.5	8.5		5.71E-13	Basic set
AHG	Levant_N	Iran_N	93.8	-2.6	8.8	19.2	11.6	9.5	9.62E-02	Basic set

Supplementary Table 7. qpADM Admixture models of the Anatolian Ceramic farmers (ACF). The proportions estimated for each ancestral source (Ref1-3) used to model the target population ACF are given. Fitting models (Pval > 0.05; and admixture proportions are feasible) are highlighted in green. When resolution is lacking to determine whether a mixture proportion is required the source population, proportion and Standard error are highlighted in lighter green. The fitting models with minimal waves of ancestry are marked in bold. The standard errors were estimated by 5 cM block jackknifing. The abbreviations of the population names are listed in Supplementary Data 2. We define the 'Basic set' of outgroup populations as *Han; Onge; Mbuti; Natufian; Kostenki14; Mala; Mixe*.

**Ancestral sources**			**Mixture proportions (%)**			**Standard errors (%)**				
**Ref1**	**Ref2**	**Ref3**	**Ref1**	**Ref2**	**Ref3**	**Ref1**	**Ref2**	**Ref3**	**Pval (rank – 1)**	**Outgroups**
**AAF**	**Levant_N**		**78.7**	**21.3**		**3.5**	**3.5**		**6.06E-01**	**Basic set**
AAF	EHG		110.3	-10.3		2.4	2.4		2.69E-02	Basic set
AAF	Iran_N		117.2	-17.2		4.6	4.6		4.21E-02	Basic set
AAF	WHG		122.9	-22.9		5.8	5.8		5.49E-02	Basic set
AAF	Levant_N	Iran_N	86.9	17.8	-4.7	9.2	5.3	4.8	5.66E-01	Basic set
**AHG**	**Levant_N**		**84.3**	**15.7**		**6.8**	**6.8**		**1.15E-01**	**Basic set**
AHG	EHG		108.0	-8.0		3.9	3.9		7.49E-02	Basic set
AHG	Iran_N		106.5	-6.5		7.0	7.0		7.65E-02	Basic set
AHG	WHG		124.1	-24.1		9.8	9.8		3.98E-01	Basic set
AHG	Levant_N	Iran_N	77.9	18.0	4.2	13.3	8.2	6.5	2.16E-01	Basic set
AHG	Levant_N	Iran_N	71.0	22.3	6.7	5.6	4.7	3.2	3.50E-01	Basic set;WHG;EHG

Supplementary Table 8. qpAdm Admixture models of Levantine early farmers. The proportions estimated for each ancestral source (Ref1-2) used to model each target population are given with their standard errors estimated by 5 cM block jackknifing. Fitting models (Pval > 0.05; and admixture proportions are feasible) are highlighted in green. When resolution is lacking to determine whether a mixture proportion is required the source population, proportion and Standard error are highlighted in lighter green. The fitting models with minimal waves of ancestry are marked in bold. The abbreviations of the population names are listed in Supplementary Data 2. We define the 'Basic set' of outgroups as: Han; Onge; Mbuti; Natufian; Kostenki14; Mala; Mixe. When Natufian was used as a source population, the present-day BedouinB Near-Eastern population was used as an outgroup in the 'Basic set' instead. The previously published individuals from Motza, Israel and 'Ain-Ghazal, Jordan [57] are grouped together and labeled as 'Levant_N'. When the published (Levant_N) and newly reported Levantine early farmers (BAJ001, KFH2) are grouped into one population they are labeled 'Levant_Neol'.

**Target**	**Ancestral sources**		**Mixture proportions (%)**		**Standard errors (%)**			**Outgroups**
	**Ref1**	**Ref2**	**Ref1**	**Ref2**	**Ref1**	**Ref2**	**Pval (rank – 1)**	
KFH2	ACF	Levant_N	-5.4	105.4	25.6	25.6	0.377	Basic set; EHG; WHG
KFH2	AAF	Levant_N	-2.6	102.6	16.5	16.5	0.313	Basic set; EHG; WHG
KFH2	AHG	Levant_N	-2.2	102.2	16.5	16.5	0.322	Basic set; EHG; WHG
KFH2	WHG	Levant_N	6.1	93.9	6.3	6.3	0.435	Basic set
**KFH2**	**ACF**	**Natufian**	**24.9**	**75.1**	**16.6**	**16.6**	**0.525**	**Basic set; EHG; WHG**
**KFH2**	**AAF**	**Natufian**	**23.3**	**76.7**	**13.5**	**13.5**	**0.622**	**Basic set; EHG; WHG**
Levant_N	ACF	BAJ001	9.8	90.2	14.5	14.5	0.625	Basic set
Levant_N	AAF	BAJ001	3.4	96.6	10	10	0.510	Basic set
Levant_N	AHG	BAJ001	-4.8	95.2	15.3	15.3	0.580	Basic set
Levant_N	WHG	BAJ001	-2.4	102.4	4.5	4.5	0.607	Basic set
Levant_N	Greece_EN	BAJ001	7.6	92.4	15.4	15.4	0.496	Basic set
**Levant_N**	**ACF**	**Natufian**	**30.4**	**69.6**	**7.2**	**7.2**	**0.575**	**Basic set; EHG; WHG**
**Levant_N**	**AAF**	**Natufian**	**21.3**	**78.7**	**6.3**	**6.3**	**0.375**	**Basic set; EHG; WHG**
**BAJ001**	**ACF**	**Natufian**	**15.8**	**84.2**	**12.5**	**12.5**	**0.128**	**Basic set; EHG; WHG**
BAJ001	AAF	Natufian	8.3	91.7	10.3	10.3	0.098	Basic set; EHG; WHG
**Levant_Neol**	**AAF**	**Natufian**	**17.8**	**82.2**	**6.4**	**6.4**	**0.288**	**Basic set; EHG; WHG**
**Levant_Neol**	**AHG**	**Natufian**	**16.3**	**83.7**	**6.7**	**6.7**	**0.074**	**Basic set; EHG; WHG**

Supplementary Table 9. Admixture models of the Iron Gates hunter-gatherers (Iron Gates HG). The proportions estimated for each ancestral source (Ref1-3) used to model Iron Gates HG population are given with their standard errors estimated by 5 cM block jackknifing. Fitting models (Pval > 0.05; and admixture proportions are feasible) are highlighted in green. When resolution is lacking to determine whether a mixture proportion is required the source population, proportion and Standard error are highlighted in lighter green. The fitting models with minimal waves of ancestry are marked in bold. The abbreviations of the population names are listed in Supplementary Data 2. We define the 'Basic set' of outgroups as: *Han; Onge; Mbuti; Natufian; Kostenki14; Mala; Mixe*. When *Natufian* was used as a source population, the present-day *BedouinB* Near-Eastern population was used as an outgroup in the 'Basic set' instead.

**Ancestral sources**			**Mixture proportions (%)**			**Standard errors (%)**				
**Ref1**	**Ref2**	**Ref3**	**Ref1**	**Ref2**	**Ref3**	**Ref1**	**Ref2**	**Ref3**	**Pval (rank-1)**	**Outgroups**
AHG	WHG	EHG	25.8	62.9	11.3	5.0	7.4	3.3	3.08E-01	Basic set
AAF	WHG	EHG	21.8	69.0	9.2	4.1	5.8	2.8	5.41E-01	Basic set
Iran_N	WHG	EHG	6.2	90.3	3.5	2.9	4.4	3.0	1.10E-02	Basic set
Natufian	WHG	EHG	11.1	78.0	10.9	2.2	4.6	3.0	5.89E-01	Basic set

Supplementary Table 10. Basal Eurasian proportion estimates. The proportions estimated for each ancestral source (Ref1-3) used to model the target population are given with their standard errors estimated by 5 cM block jackknifing. The mixture proportion of ‘Mota.SG’ listed in bold IS used as a proxy for the proportion of Basal Eurasian ancestry (α) in the target population [57]. Fitting models (Pval > 0.05; and admixture proportions are feasible) are highlighted in green. The abbreviations of the population names are listed in Supplementary Data 2. The set of outgroups used for this analysis includes: *Han; Onge; Mbuti; Natufian; Kostenki14; Mala; Mixe*. For models where Mota.SG was added to this set, the target is marked with an asterisk.

	**Ancestral sources**			**Mixture proportions (%)**			**Standard errors (%)**			
**Test**	**Ref1**	**Ref2**	**Ref3**	**Ref1**	**Ref2**	**Ref3**	**Ref1**	**Ref2**	**Ref3**	**Pval (rank-1)**
AHG	Mota.SG	WHG		**24.8**	75.2		5.5	5.5		2.72E-02
Natufian	Mota.SG	WHG		**38.5**	61.5		5.0	5.0		2.36E-01
Natufian	Mota.SG	EHG		**66.2**	33.8		3.7	3.7		8.03E-07
Natufian	Mota.SG	WHG	EHG	**36.2**	69.0	-5.2	6.2	12.4	7.7	1.16E-01
Iron_Gates_HG	Mota.SG	WHG	EHG	**1.6**	85.9	12.5	2.8	5.3	3.3	6.80E-02
Iron_Gates_HG	WHG	EHG		88.4	11.6		2.9	2.9		1.12E-01
Iron_Gates_HG*	WHG	EHG		88.6	11.4		2.9	2.9		1.57E-01

Supplementary Notes

Supplementary Note 1: Description of samples and archaeological information

Pınarbaşı – archaeological information

Pınarbaşı is situated 33.4 km southeast of Boncuklu on the eastern edge of the western Konya basin (37° 29'N, 33°02'E) at the end of the Bozdağ limestone hills, northwest of the Karadağ mountain, and represents the only excavated Epipaleolithic site on the central Anatolian plateau[1]. The site contains a series of rock shelters and caves, the most northerly of which was subjected to excavation, as Area B. The site was initially excavated in 1994-95 by Professor Trevor Watkins of Edinburgh University[2]. Excavations recommenced in 2003 under the direction of Professor Douglas Baird from the University of Liverpool and resulted in the detection and excavation of the Epipaleolithic deposits. Occupation of the site is evidenced by a long settlement sequence that commences by c. 13,500 cal BC in one of the rock shelters[1]. At the beginning of the Holocene, c. 9600 cal BC, the site saw the emergence of a sedentarising community on a small mound c. 100m west of the Epipaleolithic rock shelter[3]. The mound settlement was excavated in Areas A and D[3].

The Epipaleolithic population represented at Pınarbaşı was probably highly mobile and low density, ranging over a large area of the central Anatolian plateau and quite possibly south of the Taurus peaks in winter[1]. At Pınarbaşı they hunted local wild caprines and cattle, wetland birds and fish, with no clear evidence for any significant exploitation of local plants. Notably ancestors of the first cultivated cereals are absent[1]. Strong links to other Epipaleolithic groups especially the Natufian of the Levant but also to groups on the Mediterranean coast of Turkey have also been documented through similarities in and exchanges of material culture, especially chipped stone, obsidian and sea shell beads, as well as technological, ritual and social practices[1, 4, 5]. The 10th-9th millennium cal BC saw the development of more sedentary practices at Pınarbaşı but this

occupation lacks evidence of exploitation of wild ancestors of early cultivated plants, cultivation of cereals and legumes and animal herding[3].

Pınarbaşı - sample description

Five individuals were selected for ancient DNA analysis, skeletons ZBC and ZBD from the Epipaleolithic rock shelter and ZDS, ABM and ZAN from the open air 10th-9th millennia site (Supplementary Data 1). Only skeleton ZBC, found within Grave 13, provided sufficient genomic data. This burial was placed in an oval cut, early in the Epipaleolithic sequence at the site and thus overlaid by c. 1 m of Epipaleolithic occupation deposit and rock shatter from the rock-shelter wall. It was thus very well stratified within the site sequence. Anthropological analyses by Dr Kirsi Lorentz identified this burial as belonging to a c. 25-29 year old male[1]. The body was found fully articulated, extended in supine position, with both hands resting at the pelvis area and was lacking the cranium (Supplementary Figure 10). The presence of maxillary teeth within the grave together with the absence of evidence for major disturbance, suggests that skull removal practice characteristic of Aceramic Neolithic communities was already practised and is one feature that suggests interactions with Levantine Natufian communities where it is sporadically attested within Natufian mortuary practices[1].

Two radiocarbon dates are available for this specific articulated skeleton, ZBC. The original date of 14,209-13,121 cal BCE (2 sigma range 95.4%) (OxA 16536)[1] was confirmed with a second date of 13,646-13,284 cal BCE (2 sigma range 95.4%) (MAMS 31616) (Supplementary Table 1) from the same phalanx bone that was subjected to ancient DNA analysis. This second date, confirms the original date but usefully has a shorter range, which supports the view that this burial probably predates the Natufian or possibly overlaps with its very earliest phases, and certainly predates the Bolling-Allerod/GI 1 interstadial[1].

Boncuklu - archaeological information

Boncuklu is situated on the Konya Plain (37° 45'N 32°52'E) and lies 33.4 km northwest of Pınarbaşı and 10.2 km northeast from Çatalhöyük. The site was discovered during the archaeological surveys of the Konya Plain between 1994-2002 under the direction of Professor Douglas Baird from the University of Liverpool. Excavations began in 2006 and continue at the present time[3]. Occupation of the site is documented from 8300-7600 cal BC directly through radiocarbon dating. However, stratigraphic and material evidence suggest a slightly longer occupational span[3, 5, 6].

The Boncuklu community seems to have relied on the exploitation of wild resources to a large degree, especially wild cattle and boar, fish and wetland birds along with nuts and fruits from surrounding hill areas[3, 6]. To these resources were added small-scale cultivation of wheat, lentils and peas[3]. The chipped stone industry was microlithic, in significant contrast to broadly contemporary Levantine PPNB and northern Fertile Crescent assemblages and thus shows significant continuities with the earlier local Epipaleolithic and the earlier 10th/early 9th millennium BC community at Pınarbaşı in technological and raw material exploitation traditions[3, 6]. There is thus strong archaeological evidence of continuities from Epipapalaeolithic and early Holocene forager communities with the community at Boncuklu. Thus by 8300 cal BC it appears local foragers adopted domestic plants from areas to the south and east and fitted them into their traditional wetland exploitation practices[3]. They were presumably introduced to the region as a consequence of the far reaching and continuous interactions with neighbouring regions from the Epipaleolithic through the 10th-early 9th millennia cal BC, as also documented at earlier and contemporary Pınarbaşı[3].

The site possessed a number of sub-oval domestic buildings with mudbrick walls The Boncuklu houses underwent repeated continuous reconstruction over multiple generations in the same location, a pattern similar to other certain Aceramic Neolithic sites in the surrounding

regions, for example, to the north east at Aşıklı from 8300 cal BC[7], just to the south at Çatalhöyük from 7100 cal BC[8], in the Levant at PPNA Jericho[9] and in PPNB Tell Halula[10]. Primary inhumations were buried under the houses during their occupation, a common practice across the Near-Eastern Neolithic, but there were also primary burials and burials of deliberately disarticulated human remains including skulls in open areas between buildings. More than 37 Neolithic burials, plus 274 individual bones and 129 isolated finds of human remains have been found in the site so far[11].

Boncuklu – sample description

Skeletal samples from 31 individuals from areas H, K, M and Q were selected for genetic analysis (Supplementary Data 1), from which five, described in detail below, provided sufficient genomic data (Supplementary Data 1). Three of these burials (ZHAJ, ZHAG and ZHJ) were all articulated primary inhumations stratified within a sequence of 4 buildings in Area H, all were securely stratified under long sequences of plaster floors, two were buried during the ongoing occupation of one of the buildings. One of these, ZHJ, was directly dated by C14 to 8269-8210 cal BC in a Bayesian model[3]. ZHAJ and ZHAG were securely stratified earlier than this and thus definitively predate 8200 cal BC. ZKO was a primary fully articulated inhumation buried under the plaster floor of a building, within a stratified sequence of six buildings in Area K. It was therefore securely stratified, overlaid by a large number of plaster floors. Unpublished C14 dates from this sequence of buildings clearly indicate it falls within the main sequence dated at the site to 8300-7800 cal BC. ZMOJ is a primary inhumation, although with some elements disturbed by animal burrows, deeply stratified in a sequence of midden deposits in Area M. Currently this sequence is not directly dated, although associated artifacts suggest it overlaps with the other

dated excavation sequences in the main excavated phases of occupation at the site, within the date ranges outlined above.

ZHAJ (Area H, Grave 27). This is a primary single inhumation of a middle age adult buried in a sub-oval cut. The individual was found lying tightly flexed on their left side, positioned east-west with the head towards the west and facing north. Considerable damage from bioturbation had disturbed the arms and pelvis[11]. Initial anthropological analysis based on the skull in the absence of pelvis suggested a possible male. However, ancient DNA analysis has determined that this individual was a female (Table 1).

ZHAG (Area H, Grave 18). Grave 18 contained a double inhumation of a middle age adult female (ZHAF) and a perinatal baby (ZHAG) found in an oval cut larger than average. The adult individual (ZHAF) was found lying tightly flexed on their left side and positioned with a northeast-southwest orientation with the head towards the northwest. The perinatal individual was articulated and found with the head on top of the adult pelvis[11]. The female sex of the adult individual (genome labelled as Bon005_pub) could be confirmed by ancient DNA[12]. In the present study we could also determine the sex of the perinatal individual as a female (Table 1).

ZHJ (Area H, Grave 15). This is a primary single old adult inhumation found in a sub-oval cut. The individual was found in a crouched position lying on its right side and positioned north-south with the head orientated towards the south (Supplementary Figure 11). The bones were relatively well preserved compared with other graves although burrowing animals have destroyed parts of the skull and axial skeleton, including the left foot[11]. Morphological sexing was difficult because the remains were gracile, probably as a result of the ageing process. Ancient DNA analyses allowed us to establish that this individual was a female (Table 1).

ZKO (Area K, Grave 12). This is a single inhumation of an old adult male in an oval cut. The individual was found lying tightly flexed on their left side and orientated east-west with the

head towards the east. Scattered fragments of at least two infants (ZKM, ZKR, ZKQ, KQE), probably from earlier disturbed primary burials were also recovered from the grave fill. The bones were generally well preserved, but rodent burrowing activity caused significant disturbance to the ribs, scapula and vertebrae[11]. We were able to confirm through DNA analysis the sex of this individual as a male (Table 1).

ZMOJ (Area M, Grave 49, associated unit MAKR). A primary but heavily disturbed burial of a young adult in a sub-circular grave. The individual was orientated east-west with head to the west and facing north. The skull was found at one end of the grave and many of the other bones had been moved by animal action, so their anatomical position was not maintained[11]. We could determine through ancient DNA that this individual was male (Table 1).

Kfar HaHoresh - archaeological information

Kfar HaHoresh (32°42'13.3"N 35°16'13.3"E, 375 m above sea level) is a small, 0.75 ha, Pre-Pottery Neolithic B (PPNB) site on the western flanks of the Nazareth hills in lower Galilee, Israel. It is situated within a rock escarpment embayment in the uppermost reaches of a small tributary wadi that flows to the Jezreel valley and thence to the Mediterranean coast 25 km distant. During seventeen excavation seasons (1991-2012) a total of 500 m² were excavated[13-15]. Three principle stratigraphic phases were identified, broadly corresponding to the Early, Middle and Late PPNB, dating from ca. 8,600 cal BCE to after 7,500 cal BCE[16]. Occupation intensity increased through the sequence.

The earliest occupation is dominated by a massive, walled and lime plaster-surfaced, quadrilateral podium (Locus 1604), ca. 22 x 10 m, with at least three architectural sub-phases and a hearth molded into the earliest plastered surface[15]. Plaster curling up at the edge of the walls likely indicate the use of a mudbrick parapet (not preserved). In the center of the podium a grave

(Locus 1005) was dug into sterile sediments below the lowermost plaster surface containing a partially articulated but headless adult human male that also included remains of a herd of eight aurochs (*Bos primigenius*), likely representing a funeral feast[17, 18]. In open areas south of the podium at least two more pits containing mostly aurochs remains were documented[19], as were headless single inhumations, one articulated, the other an unusual secondary burial.

The later phases are characterized by smaller, mostly quadrilateral but also oval plaster-surfaced structures (one painted red), none exceeding 5 x 5 m, sometimes in association with one or more retaining walls on the upslope side, likely representing foundations of mudbrick retaining walls and/or parapets. The structures are accompanied by other terrace/dividing walls, platforms, cists, monoliths, postholes (seemingly non-architectural), and a numerous and diverse array of combustion features: hearths, ovens, kilns [20] and midden deposits, including knapping pits and caches[21, 22].

Later phase burials are more numerous and varied; they include single and multiple, fully articulated, with or without skulls, as well as secondary burials, skull caches and more common isolated human remains than before[15, 16, 23]. Remains of three plastered skulls were recovered, all of young adult males, one painted with cinnabar from the Taurus Mountains in southern Turkey[24]. Many graves either directly underlie architectural plastered surfaces or are covered by chalky plaster. Though including males and females and all age cohorts from neonates to elderly, the demographic profile is unusual, with an emphasis on young adult males, 20-29 years old[25]. Of note are two graves (Locus 1003 and Locus 1155 complex) under different plastered surfaces, each with minimum number of individuals (MNIs) of 17, and both containing mixtures of articulated and secondary remains (but few cranial elements), the bones having been carefully arranged[23, 26, 27]. Morphological analyses on the teeth are rather heterogeneous, but clearly show one cluster (mostly from L1003) belonging to a quite homogenous group, suggesting close

biological relations between females and sub-adults that may indicate matrilocal residence patterns[28]. Grave-goods include animal (aurochs, fox, goat and gazelle) remains as well as projectile points, sickles, ground-stone tools, marine molluscs, exotic minerals, ochre and clay tokens[29].

The fauna at Kfar HaHoresh indicate that wild ungulates (aurochs and gazelle) were preferentially selected (in contrast to coeval sites in the region), as well as evidence for increasing goat management through the sequence[30-32]. Among smaller species fox, hare, tortoise, cat, birds and fish are notable. Preservation of palaeobotanical remains is almost non-existent, though a seed of *Vicia faba* was identified.

Abundant and varied small finds categories were recovered. The huge chipped stone assemblages were made on-site using three knapping technologies - an *ad hoc* blade/flake approach, one for serial blade production from bidirectional (naviform) cores, and one for bifacial tools[14, 33]. Tools include sickle blades, projectile points, perforators, burins and chamfered items, axes and knives. Bone tools were present in some quantity. Passive ground-stone tools include querns and workslabs, while active items include numerous pounders, hammerstones, abraders, polishers, grooved items and minute polished pebbles. Abundant baked clay items include tokens and figurines, while a small ceramic assemblage was also identified[34]. Marine and freshwater molluscs are common, with most deriving from the Mediterranean though also from the Red Sea[35]. Colourful exotic minerals, in the form of lumps, pendants and beads include obsidian, malachite, amazonite, jet, bitumen and carnelian; sources range from south/central Turkey, northern Syria, the Rift valley, the Negev/Sinai, and southern Transjordan[36]. Animal and human figurines are made on clay and stone, with the only gendered items relating to phallic imagery.

Based upon its modest size, unusual and secluded setting, the lack of adjacent arable land, and the nature of the recovered finds, Kfar HaHoresh is interpreted as a local cult and funerary

locality that was probably only occupied on a periodic basis[37]. It may have served neighbouring lowland village communities, such as Yiftahel[38], situated 7 km to the northwest, or Mishmar HaEmeq, 15 km to the southwest[39].

Kfar HaHoresh – sample description

Among three petrous bones from different graves that were sampled (KFH 1-3) only KFH2 had sufficient genomic data for subsequent genetic analysis (Supplementary Data 1).

KFH2 derives from square J53 (elevation 390/400cm below datum; L1003 catalogue #1050) in the multiple grave, Locus 1003, underlying the corner of a quadrilateral plastered surface, L1001 (Supplementary Figure 12). It is the nearly complete cranium of a 0-3 year old infant, genetically identified here as a female. The same petrous bone that was used for the DNA analysis was radiocarbon dated to 7,712-7,589 cal BCE (2 sigma range 95.4%) (MAMS 30693) (Supplementary Table 1). The C14 date and stratigraphic considerations indicate the grave dates to the transition from the Middle to Late PPNB phase at the site.

Ba'ja - archaeological information

The Neolithic site of Ba'ja (35°27'45" E / 30°24'55" N) was discovered during a survey in 1983 by M. Lindner and identified as a late Pre-Pottery Neolithic site by Hans Georg K. Gebel[40]. Three soundings were carried out in 1984. Large scale excavations started in 1997 (co-directed by Bienert and Gebel) and were continued from 1999 until 2007 directed by Gebel[41]. In 2008, 2010 and 2012 special investigations at the site and in the region were carried out[42]. Besides several test units, five large areas have been excavated so far (B-South, B-North, C, D, F). In 2016, during a pilot project, new burials were discovered[41], including that of BAJ001, the

individual genetically analyzed in this study. Based on the promising results of the test phase, a new 3-years project started in 2018.

Ba‘ja is famous for its extraordinary location in a naturally secluded setting (altitude: 1140-1175 m a.s.l.), surrounded by steep slopes (Supplementary Figure 13B). Access is possible through an up to 70 m deep gorge, the *Siq al-Ba‘ja*. Though the intramontane basin on which the village rests is only about 1.5 ha large, the site is considered a *mega-site*[43], since it resembles in some aspects other sites of this phenomenon, such as Beidha[44], Basta and other middle and late Pre-Pottery Neolithic sites of the southern Levant[40].

Radiocarbon dates confirm the typological dating to the late Pre-Pottery Neolithic B (second half of the 8th millennium BCE)[45]. Preliminary analyses of archaeobotanical and archaeozoological analyses were presented by Neef (1997) and von den Driesch et al. (2004)[46]. Beside domesticated cereals (especially *Triticum dicoccum*), wild fruits such as pistachio, hawthorn, and fig were collected; charcoal analyses comprise juniper and pistachio, but no remains of oak have been discovered so far. Animal husbandry was dominated by ovicaprines, but hunting also played an important role (for meat and fur). Taken together the species suggest a year-round occupation of the site. This is corroborated by the elaborate architecture. In every trench, densely packed clusters of buildings were discovered with at least two-storeyed terraced buildings with cellar-type substructures, indicating that the whole plateau was once occupied in a pueblo-like manner[45]. “Ba‘ja’s final occupation, interrupted by at least one earthquake, is reconstructed as a densely built village without open spaces and lanes, with houses/ rooms accessible from roof tops or lower roofs, representing the settlement’s communal space”[41].

Flint industries (thoroughly studied by[42, 47]) as well as the production of various other objects, above all sandstone rings[48] and beads, provide valuable information on social identities, exchange and development supported by immaterial values. As suggested by Hans Georg Gebel

depositions of objects related to households and burials beneath floors might relate to practices of "avoidance, strengthening, fear, commodification and recommodification"[41, 49].

The dead were buried either in abandoned houses, in between houses or beneath floors. One primary burial in the most western part of the site and three collective burials had been discovered in Area C and D during earlier excavations[50-52]. Results of palaeoanthropological work were presented by Schultz et al.[53, 54]. Two new burials were discovered during the 2016 season in Area C, Room CR35 (Loci 405 and 408). These burials and further observations in adjacent rooms of Area C suggest that this area had been used as an intramural burial ground between the earliest and later architectural phases[41].

Loc. 405 is a double burial of two infants aged 0.5-1 year and 3-4 years (labeled here as BAJ001 and BAJ002 respectively). They were buried in a crouched position, squeezed in a rather small pit. BAJ001 oriented E-W slightly above BAJ002. BAJ002 was oriented W-E. The two were facing each other.

Loc. 408 is a single primary burial which is outstanding in several respects. The grave construction as well as the burial ritual was very complex (for a detailed description see[41]). Moreover, the young adult individual (labeled here BAJ003) was buried with two categories of "grave goods": seemingly personal items such as beads of various exotic raw-materials, arm rings on each upper arm still in situ and a deliberately destroyed "mace head" near the left shoulder. Additionally more objects were embedded in the grave cover.

Individual BAJ003 was lying on its left side with the legs in a crouched position. The orientation of the skeleton was SW-NE with the orientation of the face remaining unknown because the skull had fallen onto the chest, the mandible being turned upside down. Taphonomic processes indicate that there must have been a void into which the sand had penetrated only after decay and that the head was originally slightly elevated[41].

Similar to the collective burial in the same room, both graves were covered with stones slabs, which were then buried with up-to-fist sized stones and reused plaster/limestone fragments from an ancient floor.

A charcoal sample from the upper filling of the double infant burial was dated to 7027-6685 BCE (2 sigma range; MAMS 3015: 7928± 27 BP).

Out of the three sampled individuals (BAJ001 – BAJ003) only BAJ001 had sufficient genomic data for subsequent genetic analysis (Supplementary Data 1).

Ba‘ja - Sample description

The double infant burial of Locus 405 comprised two complete skeletons of young infants (BAJ001 and BAJ002). The bones are of brittle consistency, inhibiting some morphological measurements. The preservation of the bone surfaces in both skeletons is rather good.

Age at death was estimated using the dental development, lengths of long bones, development of the cranium, carpals, and vertebrae[55]. Applying these methods, age at death of the younger infant (BAJ001) was estimated between 0.5-1 year and of the older infant between 3-4 years (BAJ002).

Sex was roughly estimated by the markers of the mandible which suggested the BAJ001 individual was a male and BAJ002 a female. However, the genetic sexing determined BAJ001 to be a female (Table 1). Both individuals are of strong and short stature (When compared to the expected stature from the level of teeth development). The younger infant shows porosity and newly built bone plaques on the internal lamina of the skull, possibly being remnants of severe bleeding.

Supplementary Note 2: Genetic analysis of Neolithic Levantines

The Near-Eastern Levantine corridor, a narrow strip of land parallel to the Mediterranean Sea extending from the Sinai Peninsula to the north of Syria was one of the earliest centers of farming and cultivation[56]. Recently reported early farmer genomes from the southern Levant showed considerable genetic continuity with Epipaleolithic Natufian individuals. However, they harbored additional admixture from an Anatolian-Neolithic related gene pool[57], providing a first glimpse at the demographic history throughout the Neolithic transition in this area. However, due to the poor DNA preservation in the region, the available genomic data is mostly of low coverage and limited to a handful of sites. Therefore, additional genomes that would fill temporal and geographical gaps in the available data could shed light on the demographic structure and heterogeneity of both the Levantine hunter-gatherers and early farming Levantine populations. We compared the newly produced genome-wide data of two individuals, a ca 9,600 ya Levantine PPNB early farmer (KFH2) from the site of Kfar HaHoresh in northern Israel and another Levantine PPNB early farmer (BAJ001) from the site of Ba'ja, Jordan (Table 1), to the previously published genome-wide data of contemporaneous individuals from the sites of Motza, Israel and 'Ain-Ghazal, Jordan[57] (grouped together and labeled Levant_N, following a previous labeling system[2]). We estimated an average coverage of 0.16 and 0.75 fold with 67,535 and 254,565 covered SNPs overlapping with the Human Origins dataset for KFH2 and BAJ001 respectively. While the coverage of KFH2 exceeds our threshold for analysis, we note that it is relatively low and provides limited statistical power and resolution. We determined the genetic sex of both KFH2 and BAJ001 as females and therefore we could not estimate their nuclear contamination rate based on X chromosome. However, based on the mitochondrial

contamination estimate, both genomes were suitable for analysis with 0-6 % contamination estimates (Supplementary Table 2).

We first projected the ancient samples onto the first two dimensions of PCA (PC1, PC2) calculated for present-day west Eurasians (Materials and Methods and Fig. 1B). KFH2, BAJ001 and the published Levantine early farmers all fall in the vicinity of the Natufian cluster, shifted to the direction of Anatolian Neolithic populations, along both PC1 and PC2; among the Neolithic Levant individuals, KFH2 is further shifted from the others to this direction. In ADMIXTURE analysis (K = 10), BAJ001, KFH2 and the published Levant_N are all modeled as a mixture of a component maximized in Natufians (84.1 %, 89.8 % and 67.8 – 89.6 % for KFH2, BAJ001 and Levant_N, respectively) and a second component maximized in Mesolithic western hunter-gatherers (WHG) (Supplementary Figure 1).

We formally tested the diversity visualized on PCA and ADMIXTURE between the new and published Levantine early farmers by the *D*-statistic of the form *D*(*KFH2/BAJ001, Levant_N/KFH2; test, Mbuti*) using both ancient and present-day worldwide populations as "*test*" (Supplementary Figure 14 A-D and Supplementary Data 4). We found the new and published individuals to be symmetrically related to most test populations within our data's resolution. The only exceptions were a slight additional affinity of Levant_N with Early Neolithic individuals from the Peloponnese region in Greece[58] (labeled Greece_EN) and with KFH2 when compared to BAJ001 (D = -4.2 and -3.2 SE, respectively; Supplementary Figure 14D).

In accordance with this result, KFH2 can be modeled with qpWave as one stream of Levant_N ancestry (χ^2 p=0.473 for rank=0), whereas two-way admixture models of KFH2 with Anatolian early farmer ancestry on top of Levant_N did not fit (Supplementary Table 8). In

addition, modeling KFH2 as a two-way mixture of Levant_N and WHG lacked resolution to detect whether KFH2 has additional WHG related ancestry compared to Levant_N. Similarly, when Levant_N was modeled with additional Anatolian early farmer ancestry on top of BAJ001, the model lacked resolution to determine whether Levant_N comprised a higher proportion of Anatolian early farmer like ancestry or Early Neolthic Peloponnese one compared to BAJ001 and the two-way model of BAJ001 and WHG did not fit (Supplementary Table 8).

KFH2, BAJ001 and Levant_N could all be separately modeled as two-way mixtures of around 75 – 85 % Natufian related ancestry and the rest from Anatolian early farmer ancestry (Supplementary Table 8). This result confirms the previously reported Levantine Neolithic ancestral mixture and indicates that the here reported Kfar HaHoresh and Ba'ja individuals share a similar ancestral composition with the published Motza and 'Ain-Ghazal ones. We do not rule out the possibility that the non-significant D-statistics are due to limited statistical power of our data. Further sampling is needed to investigate the question of the genetic diversity within the Levant Neolithic populations.

The published and new Levantine early farmers could be grouped into one population (labeled Levant_Neol) and modeled as a mixture of Natufians and AHG or AAF (18.2 ± 6.4 % AHG or 21.3 ± 6.3 % AAF ancestry; Supplementary Tables 4 and 8 and Supplementary Data 4), supporting a previously reported gene flow from an Anatolian Neolithic like population to the Levantine Neolithic gene pool[57]. Moreover, we find ACF have additional genetic affinity compared to the earlier AAF that is best represented by the ancient Levantine gene pool (Fig. 2B), suggesting that the described genetic exchange between the Neolithic Levantine and Anatolian gene pools was bidirectional.

Supplementary Note 3: Admixture modeling of the Ancient Anatolian populations

While we observe a long-term persistence of the local hunter-gatherer gene pool in Anatolia throughout the Neolithic (Fig. 1C, Supplementary Table 4), PCA and formal *f*-statistics suggest that the Anatolian hunter-gatherer (AHG), Anatolian Aceramic farmers (AAF) and Anatolian Ceramic farmers (ACF) differ in affinities to certain modern and ancient populations, likely due to differences in external genetic contributions to each of these two early farmer populations. To trace the ancestral sources of these ancient Anatolian populations we used qpAdm-based admixture modeling[59] that tests and models admixture proportions from potential source populations ("reference" populations herein) without assuming an explicit phylogeny.

For estimating admixture proportions in AHG, AAF and ACF we defined a basic set of seven outgroups, comprised of the following ancient and present-day populations.

'Basic set' = Han; Onge; Mbuti; Mala; Mixe; Natufian[57]; Kostenki14[60]

These outgroups were chosen to distinguish the ancestry of the reference populations since they broadly represent the known global genetic diversity and are unlikely to harbor recent gene flow with the target or reference populations either due to geographical/temporal distance or based on their genetic clustering in ADMIXTURE and PCA analysis[59]. The modern outgroups (Han; Onge; Mbuti; Mala; Mixe) represent a global genetic variation outside west Eurasia. The Levantine Natufian[57] population (ca 12,000 years ago) and the European Upper Palaeolithic Kostenki14[60] (ca 37,000 years ago) both represent a gene pool outside of modern genetic variation. In some cases, when a reference population did not significantly contribute to the target in the attempted admixture models, it was removed from the reference set and added to the basic outgroup set in order to increase statistical power to distinguish the references.

As a prerequisite, we tested whether each set of reference populations can be distinguished by the chosen outgroups using qpWave[57]. The chosen outgroups clearly distinguished the corresponding references in all tests we performed (χ^2 $p \leq 7.70\times10^{-33}$). For both qpWave and qpAdm we use a significance level of p=0.05 for rejecting models.

To increase statistical power, individuals were grouped together under the analysis labels: AHG, AAF and ACF. To test differential affinities within the AAF individuals, we performed the *D*-statistic of the form *D (ind1, ind2; test, Mbuti)* which resulted in non-significant results for all tested pairs to the exception of individual ZHAJ that showed slightly higher affinities (-3.50 < Z < -3.01) than other AAF individuals with some Asian related populations (Supplementary Data 10).

The Anatolian hunter-gatherer (AHG)

As expected from the PCA results (Fig. 1C) and as reflected by the *D*-statistics of the form *D (AHG, pop1; pop2, Mbuti*) (Supplementary Table 3), AHG does not form a clade with Late Pleistocene or early Holocene Near-Easterners (Natufian, Levant_N or Iran_N) nor with Mesolithic hunter-gatherers from Europe (WHG and EHG). We therefore used the above populations, which are maximally differentiated in the PCA as potential sources of the AHG ancestry (Supplementary Table 5). For this analysis, Levant_N was chosen as a proxy for the Levantine late Pleistocene gene pool.

All two-way models were rejected except for the two-way admixture (χ^2 $p = 0.158$) of a Neolithic Levantine-related gene pool (48.0 ± 4.5 %; estimate ± 1 SE) and a WHG-related gene pool (52.0 ± 4.5 %; estimate ± 1 SE). A three-way model including EHG as the third source did not increase the fit in comparison to the simpler nested two-way Levant_N + WHG model (χ^2 p

= 0.717) and one with Iran_N as the third source only marginally increased the fit (χ^2 p = 0.081; 11.9 ± 6.9 %; estimate ± 1 SE).

While these results do not suggest AHG received direct gene flow from the tested sources (which are younger than AHG), they clearly support the presence of both Levantine and European hunter-gatherer related ancestries in central Anatolia during the Pleistocene.

<u>Anatolian Aceramic farmers (AAF)</u>

Inspired by the observed genetic similarity between the Anatolian hunter-gatherer and farmers as visualized in PCA and ADMIXTURE (Fig. 1C and Supplementary Figure 1) as well as the cultural continuity evidenced by the archaeological findings[1, 3], we attempted to estimate the contribution of the endogenous AHG gene pool in AAF. Furthermore, we traced potential external genetic contributions using *D*- statistics and estimated their proportion with qpAdm.

Compared to AHG, AAF have a slight excess affinity with early Holocene populations from Iran or Caucasus and with present-day south Asians, which have also been genetically linked with ancient Iranian/Caucasus ancestry[61-62] as shown by *D(AAF, AHG; test, Mbuti)* (Fig. 2A, Supplementary Figures 2 –3 and Data tables S3 – S11). We therefore attempted to model AAF using Iran_N and AHG as two source populations. We also tested other combinations of the four reference populations mentioned above (Supplementary Table 6).

Using the basic outgroup set, the two-way model of AHG and Iran_N provided a good fit but with a rather big standard error estimate of ancestry proportion (χ^2 p = 0.054; 8.6 ± 7.2% Iran_N ancestry). To increase model resolution, we added Levant_N, EHG and WHG to the outgroup set; here we found a well- consistent model with a smaller standard error estimate (χ^2 p = 0.296; 10.3 ± 3.9% Iran_N ancestry). Modeling AAF as a sister clade of AHG (one way model

without contribution from Neolithic Iranians) results in a significantly reduced fit (χ^2 p = 0.014). In the better fitting model the AHG gene pool comprises most of the AAF ancestry (89.7 ± 3.9 %), suggesting a high degree of genetic continuity in central Anatolia from the Epipaleolithic to the Neolithic past the emergence of farming.

Our results also suggest that the additional Neolithic Iran or Caucasus related ancestry (10.3 ± 3.9 %) diffused into central Anatolia during the same 5,000-year period, although for now we cannot narrow it down further due to lack of ancient genomes between AHG and AAF. Genome-wide data from additional AHG and AAF individuals could also help to increase our resolution and more accurately quantify the differences in ancestry between the two populations.

Anatolian Ceramic farmers (ACF)

Using a similar approach as for AAF, we estimated the contribution of the AAF gene pool in ACF and used *D*-statistics to detect potential external genetic contributions and estimate their proportion.

ACF share excess affinity with the early Holocene Levantines compared with AAF, as shown by positive *D (ACF, AAF; test, Mbuti* when "test" has Levantine related ancestry*)* (Fig. 2B, Supplementary Figures 4-5 and Supplementary Data 3). When the "test" populations are ancient Iran/Caucasus related populations and contemporary South Asians excess allele sharing with ACF is not observed (Z < 1.3).

We can model ACF as a mixture of Neolithic Levantines and AAF (χ^2 p = 0.606; Supplementary Table 7). All the other tested models with AAF as a source are either rejected or produce infeasible proportions. When replacing AAF with AHG as the source population the three-way model with Levant_N and Iran_N as additional sources works well, confirming the

two sources of gene flow entering central Anatolia between the Epipaleolithic and the Ceramic Neolithic. The two-way model with only AHG and Levant_N also fits well (χ^2 p = 0.115; table S7). However, this is likely due to limited power of our data to detect such a small contribution in a complex three-way model scenario.

When ACF is modeled by AAF and Levant_N, the AAF gene pool still comprises more than 3/4 of the ancestry in ACF (78.7 ± 3.5 %), suggesting that the hunter-gatherer gene pool persisted in the region for at least 2,000 years more and indicating limited influence from external gene pools during the Neolithic.

Supplementary Note 4: Investigating genetic links between Near-Eastern and European hunter-gatherers

The Anatolian Epipaleolithic hunter-gatherer (AHG) and the Mesolithic European hunter-gatherers (WHG and EHG) show a considerable degree of genetic differentiation in PCA (Fig. 1C). Nonetheless, central Anatolia geographically connects Europe to the Near East and with major climatic changes affecting the region during the last glaciation[63] it is not unlikely that Anatolia was the ground for East and West genetic exchange during the Palaeolithic. A recent study reported an affinity between modern Near-Easterners and European hunter-gatherers post-dating 14,000 years ago compared to earlier ones[64]. With ancient genetic data available, we could directly compare the Near-Eastern hunter-gatherers (AHG and Natufian; labeled "*Near-Eastern HGs"*) and the European hunter-gatherers by *D*(*European HG, Kostenki14; AHG/Natufians, Mbuti/ Altai_published.DG*). We used the 37 thousand-year-old *Kostenki14*[60, 64] individual which is the oldest available European genome with genetic affinity to later European hunter-gatherers as a base line representing European HG pre-dating 14,000 years ago. This statistic resulted in significantly positive values for almost all individuals post-dating 14,000 years ago (*"later European HG*") when positioned in *"European HG"* while for earlier ones (*"earlier European HG*") the statistic was less positive on average and reached significance in only some individuals (Fig. 3A and Supplementary Data 5). These results suggest increased genetic affinity of later European HGs with the Near-Eastern HGs compared to the earlier ones as previously observed for modern Near-Eastern populations.

Interchanging the central African *Mbuti* with the Altai Neanderthal (*Altai_published.DG*) as an outgroup did not significantly alter the results, confirming that the observed affinities are not

caused by differing levels of Neanderthal ancestry in the tested hunter-gatherers (Supplementary Data 5).

One particular population among the later European HGs, the recently reported Mesolithic hunter-gatherers from the Balkan peninsula ('Iron Gates HG')[58], shows the most allele sharing with AHG in *D*(*Iron_Gates_HG, European HGs; AHG, Mbuti/Altai*; Fig. 3A, Supplementary Figures 6-7 and Supplementary Data 5). The Iron Gates HG population was previously modeled as a mixture of WHG, EHG and a third unknown ancestral component[58].

The geographic location of the ' Iron Gates' site within the natural corridor connecting the Near East, through Anatolia, with continental Europe as well as the genetic affinities observed in the above *D-statistic*, motivated us to consider Near Eastern HGs as potential sources for the Iron Gates ancestry. We modeled Iron Gates HG as a three-way mixture of Near-Eastern HGs or the Iran_N population (used as a proxy for Iranian hunter-gatherer ancestry) (Supplementary Table 9). The model in which Iran_N is used as the third source population was rejected. However, we can model the Iron Gates HG as a three-way mixture of AHG or Natufian (25.8 ± 5.0 % or 11.1 ± 2.2 % respectively), WHG (62.9 ± 7.4 % or 78.0 ± 4.6 % respectively) and EHG (11.3 ± 3.3 % or 10.9 ± 3 % respectively) (Supplementary Tables 4 and 9).

It should be noted that the published individuals from the Iron Gates region date several millennia later than AHG and include individuals that have been reported to be migrants from Anatolia showing northwestern Anatolian Neolithic-like ancestry[58]. We excluded from our analysis the outlier individuals showing the above Anatolian farmer ancestry to avoid signals related to Neolithic interactions that postdate the formation of the observed Pleistocene genetic link between the Near East and Europe (Supplementary Data 2).

We tested whether a model in which a gene flow from a Near-Eastern ancestry is introduced into the ancestors of Iron Gates could sufficiently explain the excess affinity we observe between the two populations. For this purpose, we exploited the fact that Near-Eastern populations harbor a Basal Eurasian ancestry component (α) which is undetectable in European hunter-gatherers[64]. Therefore the Basal Eurasian ancestry could serve as a marker for Near-Eastern gene flow. We assessed the Basal Eurasian ancestry proportion by following a previously described approach of *qpAdm* modeling[57]. This framework relies on the basal phylogenetic position of both the Basal Eurasian ghost population and an African reference (the ancient Ethiopian Mota genome[65]) relative to other non-Africans. Therefore, by using a set of outgroups that includes eastern non-African populations (Han; Onge; Papuan) and Upper Palaeolithic Eurasian genomes (Ust_Ishim[66]; Kostenki14; Malta_cluster[67]) but neither west Eurasians with detectable basal Eurasian ancestry nor Africans, the mixture proportion computed for Mota (α) can be used indirectly to estimate the Basal Eurasian mixture proportion of west Eurasian populations (Supplementary Table 10).

We estimated α to be 24.8 ± 5.5 % in AHG (Fig. 3b and Supplementary Table 9) and 38.5 ± 5.0 % for Natufian, which is consistent with previous estimates. If we assume an Anatolia to Europe gene flow, we can use our estimate for AHG derived ancestry in the Iron Gates HG to calculate the expected proportion of Basal Eurasian ancestry in Iron Gates HG (% AHG in Iron Gates HG) × (α in AHG) resulting in an expected α of 6.4 %. Yet, we could model Iron Gates HG without any Basal Eurasian ancestry or when forcing "Mota" into the model, as comprising a non-significant 1.6 ± 2.8 % (Supplementary Table 10), suggesting that it is unlikely that unidirectional gene flow from the Near East to Europe alone can account for the Iron Gates HG and the Near-Eastern HG affinity. We propose a plausible scenario in which a genetic exchange

between populations ancestral to southeastern Europeans of the early Holocene and Anatolians of the late glacial occurred before 15,000 years ago (the age of AHG).

Supplementary Note 5: Mitochondrial DNA analysis

The Anatolian hunter-gatherer (AHG)

AHG (The Pinarbaşi Epipaleolithic individual ZBC) displays 31 polymorphisms from the rCRS and can be confidently assigned to mitochondrial haplogroup K2b.

Haplogroup K2 is a sub-clade of the major haplogroup K, which according to Maximum Likelihood estimates based on complete mtDNA sequences arose ~25-29 kya during the cooling period preceding the Late Glacial Maximum[68, 69]. Bayesian estimates using several internal calibration points within haplogroup U have however provided a more recent date of ~18.5 ka (14.5-23.3 ka, 95% CI)[69, 70]. Subclade K2b detected in Pinarbaşi has been dated by Maximum Likelihood method to the Late Glacial ~17–18 ka[69]. To date, K2 and its basal sub-clusters K2a, K2b and K2c have been almost exclusively detected in modern-day Europeans, which was used as an argument for a European origin of the whole sub-clade and recent back-migration from Europe into the Near East to explain the spurious presence of K2 haplotypes in the Near East[69]. However, no K2 haplogroups have been found in pre-Neolithic Europe[70] and so far only one ancient sample with haplogroup K2b has been reported in a Corded Ware individual from Esperstedt (Germany, 2500-2050 BCE)[71]. Most modern DNA reported mitogenomes belong to sub-clusters K2b1 or in less frequency to K2b2 within K2, with just two sequences at the root of K2.

While the Pinarbaşi individual postdates the average time node of sub-cluster K2 by a couple of millennia, the presence of haplogroup K2b in Epipaleolithic Anatolia raises the possibility that this sub-clade could have a Near-Eastern origin. Interestingly, the Pinarbaşi individual lacks one of the terminal mutations characteristic of the K2b, a transition at position 14067. While this could represent a back-mutation event, it is also possible that it mutation

emerged in the Near East after 13,000 BCE and was carried into Europe afterwards. The lack of other contemporaneous representatives of K2b does not allow distinguishing between both possibilities.

The Anatolian Aceramic farmers (AAF) and the Levantine early farmers

Boncuklu individuals **ZHAJ, ZHJ** and **ZKO** all display the 19 diagnostic mutations of mitochondrial haplogroup U3. Individual ZKO shows four extra differences from rCRS, including an insertion in the HVII poly-C stretch. Individuals ZHAJ and ZHJ share the same mitochondrial haplotype, one mutational step away from ZKO (+4820A).

According to Maximum Likelihood estimates of modern mtDNA haplotype diversity, haplogroup U3 originated in the Near East during the Upper Palaeolithic ca. 32ka[68, 72]. In modern populations this haplogroup is primarily found in the Near East and the Caucasus, while it is present in lower frequency or even absent in western European populations[68, 72].

The oldest report of haplogroup U3 corresponds to Boncuklu (this publication,[12]). In the Near East it is also present in two Ceramic Neolithic individuals (ACF) from Barçin in the Marmara region dated back to 6500-6000 cal BCE[71], but it is absent in Tepecic Çiftik (5500-7800 cal BCE) and in contemporaneous PPNB-PPNC populations from the southern and northern Levant[12,57,73]. The presence of this haplogroup in Aceramic and Ceramic Anatolian Neolithic is in agreement with the genetic continuity between Anatolian pre-pottery and pottery Neolithic inferred from whole genome analyses.

In the Early European Neolithic haplogroup U3 only appears in two individuals belonging to the Starçevo (Hungary) and LBK (Germany) archaeological cultures[74]. In Europe it is otherwise detected in the Middle Neolithic Salzmünde culture in Germany (N= 5) and in the

Middle, Late Neolithic and Chalcolithic periods in Spain (N=5) [57, 75-78]. Our results are in agreement with a concomitant spread of a few members of this haplogroup with the Neolithic, which however did not have a substantial demographic impact due to genetic drift.

Boncuklu individual **ZHAG** belongs to the subclade N1a1a1 within mitochondrial haplogroup N1a. The oldest report of this haplogroup corresponds also to Boncuklu (this publication, [7]) and as described for haplogroup U3, there are no contemporaneous parallels of subclade N1a1a1 in the PPNB Levantine populations whereas it was reported in later Ceramic Anatolian populations (Barçin and Mentese)[75]. Sub-clade N1a1a1 and its derived cluster N1a1a1a (+16320T) are ubiquitously present in considerable frequencies in Early Neolithic European cultures (Starčevo, LBK, Epicardial), probably as a result of a founder effect following the spread of the Neolithic from Anatolia[59,74].

Individual **KFH2** from the PPNB archaeological site of Kfar Hahoresh is also classified as N1a, albeit from the sub-branch N1a1b. The present SNPs together with the absence of the five diagnostic positions leading to the more widely distributed sub-branch N1a1b1 places this haplotype at the root of N1a1b. Therefore, KFH2 represents the first reported prehistoric member of the N1a1b node that, according to modern phylogeographic mitochondrial data, originated ~28ka most probably in the Near East[79-80]. It is important to note that four of the diagnostic positions of the haplogroup are not covered and an additional 14 have a coverage ≤5 (Supplementary Data 6).

Individual **BAJ001** from the PPNB archaeological site of Ba'ja in Jordan harbours all the diagnostic SNPs characteristic of haplogroup N1b1a with the exception of mutation 1703, plus one extra transition in position 16519 and an extra C insertion in the HVRII poly-C tract. A back-mutation in position 1703, together with a T insertion in 455 and a transition in 8084,

define the sub-branch N1b1a1 within N1b1a. The absence of 1703 in BAJ001 therefore suggests that this back-mutation emerged before 9,000 BP and preceded the other two substitutions.

Haplogroup N1b is extremely rare in Neolithic and post-Neolithic Near East and Europe, and has been reported so far in just two ancient individuals, one belonging to the sub-clade N1b2 from Ivanovo, Bulgaria, dated back to the Middle Chalcolithic (4,725-4,605 cal BCE)[58] and interestingly, one classified also as N1b1a in the Anatolian Ceramic Neolithic site of Barcin (6500-6200 BCE)[75]. In modern populations, haplogroup N1b1 is found primarily in the Near East, with minor branches in Europe and North Africa. It reaches maximum frequencies in the southern Levant and in Ashkenazi Jewish groups[80]. Sub-cluster N1b1a has been dated to ~13-14ka[80]. In the same study, two scenarios of expansion have been postulated based on HVRI data: 1. during the Neolithic or 2. during the Late Glacial period[80]. The presence of this sub-clade in Early Neolithic Ba'ja together with its scarceness during and after the Neolithic are more in agreement with the latter.

Boncuklu individual **ZMOJ** can be assigned to mitochondrial haplogroup K1a, the SNPs found in this sample place it at the root of this sub-clade. Until recently, haplogroup K as a whole had only been detected among farmers, however recent analyses have reported haplogroup K1 in eleven hunter-gatherer individuals, two from the Mesolithic site of Theopetra in Greece (7,605-7,529 cal BCE and 7,288-6,771 cal BCE)[81], one from Măgura Buduiasca in Romania (6061-5985 cal BCE) and eight from several sites across the Iron Gates region in Romania and Serbia (ca. 5800-9000 cal BCE)[58]. Moreover one hunter-gatherer from Satsurblia in Georgia (11,430-11,180 cal BCE) carried haplogroup K3[82].

With the exception of one individual from Padina (6,061-5,841 cal BCE) with admixed hunter-gatherer and Anatolian Neolithic ancestry, none of these hunter-gatherers have been

classified as belonging to sub-cluster K1a (K3, K1, K1c and K1f). Therefore, current evidence restricts this clade to Neolithic and post-Neolithic Near-Eastern and European individuals. With the exception of individual ZHF from Boncuklu, who also carries the root haplotype of sub-clade K1a[12], Early farmers from the Levant and Anatolia belong to derived K1a sub-lineages (mainly K1a18, K1a2, K1a3, K1a4 and K1a12). The whole cluster reaches a high frequency (33%) among Anatolian ceramic farmers (Barcin, Mentese and Tepeçic Ciftik)[12, 75] and it is present in almost all early Neolithic cultures at frequencies between 10 and 20%[59,74, 76, 83]. According to modern phylogeographic studies on the diversity and distribution of K1a mitotypes, the coalescence age of K1a has been estimated in ~20 ka[69]. The absence of pre-farming representatives of K1a in Europe point out a more probable Near-Eastern origin for this sub-clade, and the spread of the Neolithic as the main source of its dispersal and diversification.

Supplementary References

1. Baird, D. *et al*. Juniper smoke, skulls and wolves' tails. The Epipalaeolithic of the Anatolian plateau in its South-west Asian context; insights from Pınarbaşı. *Levant* **45**, 175-209 (2013).

2. Watkins, T. Excavations at Pinarbasi: The early stages. *On the Surface: Ç atalhoyuk* **1995**, 47-57 (1993).

3. Baird, D. *et al*. Agricultural origins on the Anatolian plateau. *Proc. Natl. Acad. Sci. U. S. A.* **115**, E3077-E3086 (2018).

4. Baird, D. Pınarbaşı; from Epipalaeolithic campsite to sedentarising village in central Anatolia. *The Neolithic in Turkey* **3**, 181-218 (2012).

5. Baird, D. The Late Epipaleolithic, Neolithic, and Chalcolithic of the Anatolian Plateau, 13,000–4000 BC. *A companion to the archaeology of the Ancient Near East*, 431-465 (2012).

6. Baird, D., Fairbairn, A., Martin, L. & Middleton, C. in Archaeology and Art Publications. *The Boncuklu Project: the origins of sedentism, cultivation and herding in central Anatolia* (2012).

7. Özbaşaran, M. Aşıklı. *The Neolithic in Turkey* **3**, 135-158 (2012).

8. Hodder, I. Çatalhöyük: the leopard's tale: revealing the mysteries of Turkey's ancient 'town'. *Thames & Hudson* (2006).

9. Byrd, B. F. Reassessing the emergence of village life in the Near East. *Journal of Archaeological Research* **13**, 231-290 (2005).

10. Kuijt, I., Guerrero, E., Molist, M. & Anfruns, J. The changing Neolithic household: Household autonomy and social segmentation, Tell Halula, Syria. *Journal of Anthropological Archaeology* **30**, 502-522 (2011).

11. Pearson, J. in Boncuklu; first farmers in central Anatolia and the antecedents of Çatalhöyük. From foragers to farmers in central Anatolia, D. Baird, A. Fairbairn, Eds. (forthcoming), vol. 1.

12. Kılınç, G. M. *et al*. The demographic development of the first farmers in Anatolia. *Current Biology* **26**, 2659-2666 (2016).

13. Goring-Morris, A. N. A PPNB settlement at Kfar Hahoresh in lower Galilee: A preliminary report of the 1991 season. *Journal of the Israel Prehistoric Society* **24**, 77-101 (1991).

14. Goring-Morris, A. N., Goren, Y., Horwitz, L. K., Bar-Yosef, D. & Hershkovitz, I. Investigations at an early Neolithic settlement in the lower Galilee: Results of the 1991 season at Kefar Hahoresh. *'Atiqot* **27**, 37-62 (1995).

15. Goring-Morris, A. N. *et al*. The 2007–8 excavation seasons at pre-pottery Neolithic B Kfar HaHoresh, Israel. *Antiquity* **82**, 1151ff (2008).

16. Goring-Morris, N. in *Life in Neolithic farming communities* 103-136 (Springer, 2002).

17. Horwitz, L. K. & Goring-Morris, N. Animals and ritual during the Levantine PPNB: a case study from the site of Kfar Hahoresh, Israel. *Anthropozoologica* **39**, 165-178 (2004).

18. Goring-Morris, N. & Horwitz, L. K. Funerals and feasts during the Pre-Pottery Neolithic B of the Near East. *antiquity* **81**, 902-919 (2007).

19. Meier, J. S., Goring-Morris, A. N. & Munro, N. D. Depositional histories of faunal remains from the Neolithic cultic site of Kfar HaHoresh, Israel. *Journal of Anthropological Archaeology* **48**, 233-249 (2017).

20. Goren, Y. & Goring-Morris, A. Early pyrotechnology in the Near East: Experimental lime-plaster production at the Pre-Pottery Neolithic B site of Kfar HaHoresh, Israel. *Geoarchaeology* **23**, 779-798 (2008).

21. Barzilai, O. & Goring-Morris, A. N. Bidirectional blade and tool caches and stocks in the PPNB of the southern Levant. *Technical Systems and Near Eastern PPN Communities*, 227-294 (2007).

22. Davidzon, A. & Goring-Morris, N. Knapping in the graveyard: a refitted naviform sequence from Kfar HaHoresh, Lower Galilee, Israel. *Systemes techniques et communautes du Neolithique preceramique au Proche-Orient Technical Systems and Near Eastern PPN Communities.Antibes: Editions APDCA*, 295-309 (2007).

23. Goring-Morris, A. N. in *Life, death and the emergence of differential status in the Near Eastern Neolithic: evidence from Kfar HaHoresh, Lower Galilee, Israel* (2005).

24. Goren, Y., Goring-Morris, A. N. & Segal, I. The technology of skull modelling in the Pre-Pottery Neolithic B (PPNB): regional variability, the relation of technology and iconography and their archaeological implications. *Journal of Archaeological Science* **28**, 671-690 (2001).

25. Eshed, V., Hershkovitz, I. & Goring-Morris, A. N. A re-evaluation of burial customs in the Pre-Pottery Neolithic B in light of paleodemographic analysis of the human remains from Kfar HaHoresh, Israel. *Paléorient*, 91-103 (2008).

26. Goring-Morris, A. N. *et al*. The 1997 season of excavations at the mortuary site of Kfar HaHoresh, Galilee, Israel. *Neo-lithics* **3**, 1-4 (1998).

27. Hershkovitz, I. *et al*. Remedy for an 8500 year-old plastered human skull from Kfar HaHoresh, Israel. *Journal of Archaeological Science* **22**, 779-788 (1995).

28. Alt, K. W., Benz, M., Vach, W., Simmons, T. L. & Goring-Morris, A. N. Insights into the social structure of the PPNB site of Kfar HaHoresh, Israel, based on dental remains. *PloS one* **10**, e0134528 (2015).

29. Simmons, T., Goring-Morris, N. & Horwitz, L. K. IN THE PRE-POTTERY NEOLITHIC B Mortuary COMPLEX AT KFAR HAHORESH, ISRAEL. *Faces from the Past: Diachronic Patterns in the Biology of Human Populations from the Eastern Mediterranean: Papers in Honour of Patricia Smith* **1603**, 100 (2007).

30. Makarewicz, C. A., Horwitz, L. K. & Goring-Morris, A. N. Local adoption of animal husbandry in the Southern Levant: an isotopic perspective from the Pre-Pottery Neolithic B funerary site of Kfar HaHoresh. *Environmental Archaeology* **21**, 199-213 (2016).

31. Meier, J. S., Goring-Morris, A. N. & Munro, N. D. Provisioning the Ritual Neolithic Site of Kfar HaHoresh, Israel at the Dawn of Animal Management. *PloS one* **11**, e0166573 (2016).

32. Meier, J. S., Goring-Morris, A. N. & Munro, N. D. Aurochs bone deposits at Kfar HaHoresh and the southern Levant across the agricultural transition. *antiquity* **91**, 1469-1483 (2017).

33. Barzilai, O. & Goring-Morris, A. N. Bidirectional blade production at the PPNB site of Kfar HaHoresh: the techno-typological analysis of a workshop dump. *Paléorient*, 5-34 (2010).

34. Biton, R., Goren, Y. & Goring-Morris, A. N. Ceramics in the Levantine Pre-Pottery Neolithic B: evidence from Kfar HaHoresh, Israel. *Journal of Archaeological Science* **41**, 740-748 (2014).

35. Mayer, D. E. B. Shell ornaments and artifacts in Neolithic Cyprus and correlations with other Mediterranean regions. *Quaternary International* (2017).

36. Bar-Yosef Mayer, D. E. & Porat, N. Green stone beads at the dawn of agriculture. *Proc. Natl. Acad. Sci. U. S. A.* **105**, 8548-8551 (2008).

37. Birkenfeld, M. & Goring-Morris, A. Out of sight: The role of Kfar HaHoresh within the PPNB landscape of the lower Galilee, Israel. *Archaeological Review from Cambridge* **30**, 95-14 (2015).

38. Garfinkel, Y. *et al*. in *The pre-pottery Neolithic B village of Yiftahel: The 1980s and 1990s excavations* (Hebrew University of Jerusalem, Jerusalem, Israel, 2012).

39. Barzilai, O. & Getzov, N. The 2010 excavation season at Mishmar Ha'emeq in the Jezreel Valley. *Neolithics* **1**, 19-22 (2011).

40. Bienert, H. & Gebel, H. G. K. Summary on Ba'ja 1997, and insights from the later seasons. *Central Settlements in Jordan. Studies in Early Near Eastern Production, Subsistence, and Environment* **5**. *Ex oriente, Berlin*, 119-144 (2004).

41. Gebel, H. G. K. *et al*. Household and Death. Preliminary results of the 2016 11th Season at Late PPNB Ba'ja, Southern Jordan. *Neo-Lithics* **1/17**, 18-36 (2017).

42. Purschwitz, C. Die lithische Ökonomie von Feuerstein im Frühneolithikum der Größeren Petra-Region. *Studies in Early Near Eastern Production, Subsistence, and Environment* **19.** *Ex Oriente, Berlin* (2017).

43. Gebel, H. G. K. Central to what? Remarks on the Settlement Patterns of the LPPNB Mega-Sites in Jordan. *Central Settlements in Jordan. Studies in Early Near Eastern Production, Subsistence, and Environment* **5**. *Ex Oriente, Berlin*, 1-20 (2004).

44. Byrd, B. F. *Early village life at Beidha, Jordan: Neolithic spatial organization and vernacular architecture: the excavations of Mrs. Diana Kirkbride-Helbæk* (Oxford University Press Oxford, 2005).

45. Kinzel, M. Am Beginn des Hausbaus: Studien zur PPNB-Architektur von Shkārat Msaied und Ba'ja in der Petra-Region, Südjordanien. *Studies in Early Near Eastern Production, Subsistence, and Environment* **17.** *Ex Oriente, Berlin* (2014).

46. Von den Driesch, A., Cartajena, H. Manharth, The Late PPNB site of Ba'ja, Jordan: the faunal remains (1997 season). *Central Settlements in Neolithic Jordan. Studies in Early Near Eastern Production, Subsistence, and Environment* **5**. *Ex Oriente, Berlin* 271-288 (2004).

47. Purschwitz, C. Ba‘ja 2012: Abiotic resources and Early Neolithic raw material procurement in the Greater Petra area (ARGPA). Research aims and first results. *Neo-Lithics* **1/13**, 3-10 (2013).

48. Michiels, T., Al-Souliman, A. S. & Gebel, H. G. K. Stage 3 manufacturing traces of the Ba‘ja LPPNB sandstone rings. *Neo-lithics* **2/12**, 41-50 (2012).

49. Gebel, H. G. K. Commodification and the formation of early Neolithic social identity. The issues as seen from the southern Jordanian Highlands. The principle of sharing.Segregation and construction of social identities at the transition from foraging to farming. *Studies in Early Near Eastern Production, Subsistence, and Environment* **14**, 35-80 (2010).

50. Gebel, H. G. K., Hermansen, B. & Kinzel, M. Ba'ja 2005: A Two-Storied Building and Collective Burials. Results of the 6th Season of Excavation. *Neo-Lithics* **1**, 12-19 (2006).

51. Gebel, H. G. K. & Hermansen, B. D. LPPNB Ba‘ja 2001. A Short Note. *Neo-lithics* **2/01**, 15-20 (2001).

52. Gebel, H. G. K. & Hermansen, B. D., Ba‘ja 2003. Summary of the 5th season of excavation. *Neo-Lithics* **2/04**, 15-18 (2004).

53. Schultz, M., Schmidt-Schultz, T. H., Gresky, J., Kreutz, K. & Berner, M. *Die Menschen von Basta und Ba’ja im akeramischen Neolithikum.* In: B. Salje,N. Riedl, G. Schauerte and R.-B. Wartke (eds.), *Gesichter des Orients. 10000 Jahre Kunst und Kultur aus Jordanien*. *Mainz: Verlag Philipp von Zabern* 57-60 (2004).

54. Schultz, M., Schmidt-Schultz, T. H., Gresky, J., Kreutz, K. & Berner, M. Morbidity and mortality in the late PPNB populations from Basta and Ba'ja (Jordan). *BAR International Series.* **1603**, 82 (2007).

55. Beck, L. A. Standards for data collection from human skeletal remains. Edited by Jane E. Buikstra and Douglas H. Ubelaker. 272 pp. Fayetteville: Arkansas Archeological Survey Research Series No. 44, 1994. *Am. J. Hum. Biol.* **7**, 672-672 (1995).

56. Zohary, D., Hopf, M. & Weiss, E. in *Domestication of Plants in the Old World: The origin and spread of domesticated plants in Southwest Asia, Europe, and the Mediterranean Basin* (Oxford University Press on Demand, 2012).

57. Lazaridis, I. *et al*. Genomic insights into the origin of farming in the ancient Near East. *Nature* **536**, 419-424 (2016).

58. Mathieson, I. *et al*. The Genomic History of Southeastern Europe. **555**, 197-203 *Nature* (2018).

59. Haak, W. *et al*. Massive migration from the steppe was a source for Indo-European languages in Europe. *Nature* **522**, 207-211 (2015).

60. Seguin-Orlando, A. *et al*. Paleogenomics. Genomic structure in Europeans dating back at least 36,200 years. *Science* **346**, 1113-1118 (2014).

61. Broushaki, F. *et al*. Early Neolithic genomes from the eastern Fertile Crescent. *Science* **353**, 499-503 (2016).

62. Narasimhan, V. M. *et al*. The Genomic Formation of South and Central Asia. *bioRxiv*, 292581 (2018).

63. Gamble, C., Davies, W., Pettitt, P. & Richards, M. Climate change and evolving human diversity in Europe during the last glacial. *Philos. Trans. R. Soc. Lond. B. Biol. Sci.* **359**, 243-53; discussion 253-4 (2004).

64. Fu, Q. *et al*. The genetic history of Ice Age Europe. *Nature* **534**, 200-205 (2016).

65. Gallego Llorente, M. *et al*. Ancient Ethiopian genome reveals extensive Eurasian admixture throughout the African continent. *Science* **350**, 820-822 (2015).

66. Fu, Q. *et al*. Genome sequence of a 45,000-year-old modern human from western Siberia. *Nature* **514**, 445-449 (2014).

67. Raghavan, M. *et al*. Upper Palaeolithic Siberian genome reveals dual ancestry of Native Americans. *Nature* **505**, 87-91 (2014).

68. Behar, D. M. *et al*. A “Copernican” reassessment of the human mitochondrial DNA tree from its root. *The American Journal of Human Genetics* **90**, 675-684 (2012).

69. Costa, M. D. *et al*. A substantial prehistoric European ancestry amongst Ashkenazi maternal lineages. *Nature communications* **4**, 2543 (2013).

70. Pereira, L. *et al*. Population expansion in the North African late Pleistocene signaled by mitochondrial DNA haplogroup U6. *BMC evolutionary biology* **10**, 390 (2010).

71. Posth, C. *et al*. Pleistocene mitochondrial genomes suggest a single major dispersal of non-Africans and a Late Glacial population turnover in Europe. *Current Biology* **26**, 827-833 (2016).

72. Soares, P. *et al*. The archaeogenetics of Europe. *Current Biology* **20**, R174-R183 (2010).

73. Fernández, E. *et al*. Ancient DNA analysis of 8000 BC near eastern farmers supports an early Neolithic pioneer maritime colonization of Mainland Europe through Cyprus and the Aegean Islands. *PLoS genetics* **10**, e1004401 (2014).

74. Szecsenyi-Nagy, A. *et al*. Tracing the genetic origin of Europe's first farmers reveals insights into their social organization. *Proc. Biol. Sci.* **282**, 10.1098/rspb.2015.0339 (2015).

75. Mathieson, I. *et al*. Genome-wide patterns of selection in 230 ancient Eurasians. *Nature* **528**, 499-503 (2015).

76. Brandt, G. *et al*. Ancient DNA reveals key stages in the formation of central European mitochondrial genetic diversity. *Science* **342**, 257-261 (2013).

77. Szecsenyi-Nagy, A. *et al*. The maternal genetic make-up of the Iberian Peninsula between the Neolithic and the Early Bronze Age. *Scientific reports* **7**, 15644 (2017).

78. Gómez-Sánchez, D. *et al*. Mitochondrial DNA from El Mirador cave (Atapuerca, Spain) reveals the heterogeneity of Chalcolithic populations. *PLoS One* **9**, e105105 (2014).

79. Olivieri, A. *et al*. Mitogenomes from two uncommon haplogroups mark late glacial/postglacial expansions from the near east and Neolithic dispersals within Europe. *PloS one* **8**, e70492 (2013).

80. Fernandes, V. *et al*. The Arabian cradle: mitochondrial relicts of the first steps along the southern route out of Africa. *The American Journal of Human Genetics* **90**, 347-355 (2012).

81. Hofmanova, Z. *et al*. Early farmers from across Europe directly descended from Neolithic Aegeans. *Proc. Natl. Acad. Sci. U. S. A.* **113**, 6886-6891 (2016).

82. Jones, E. R. *et al*. Upper Palaeolithic genomes reveal deep roots of modern Eurasians. *Nat. Commun.* **6**, 8912 (2015).

83. Olalde, I. *et al*. A common genetic origin for early farmers from Mediterranean Cardial and Central European LBK cultures. *Mol. Biol. Evol.* **32**, 3132-3142 (2015).
