# Supplementary material for: Late Pleistocene human genome suggests a local origin for the first farmers of central Anatolia
Source: Nat Commun. 2019 Mar 19;10:1218. doi: 10.1038/s41467-019-09209-7 (PMC6425003; doi:10.1038/s41467-019-09209-7)
Supplement: Supplementary file 3 — Reporting Summary [file 41467_2019_9209_MOESM3_ESM.pdf]

nature research

Corresponding author(s): Eva Fernández-Domínguez, Choongwon Jeong and Johannes Krause

Last updated by author(s): Jan 22, 2019

Reporting Summary

Nature Research wishes to improve the reproducibility of the work that we publish. This form provides structure for consistency and transparency in reporting. For further information on Nature Research policies, see Authors & Referees and the Editorial Policy Checklist.

Statistics

For all statistical analyses, confirm that the following items are present in the figure legend, table legend, main text, or Methods section.

n/a	Confirmed	
☐	☒	The exact sample size (*n*) for each experimental group/condition, given as a discrete number and unit of measurement
☒	☐	A statement on whether measurements were taken from distinct samples or whether the same sample was measured repeatedly
☐	☒	The statistical test(s) used AND whether they are one- or two-sided<br>*Only common tests should be described solely by name; describe more complex techniques in the Methods section.*
☒	☐	A description of all covariates tested
☐	☒	A description of any assumptions or corrections, such as tests of normality and adjustment for multiple comparisons
☐	☒	A full description of the statistical parameters including central tendency (e.g. means) or other basic estimates (e.g. regression coefficient) AND variation (e.g. standard deviation) or associated estimates of uncertainty (e.g. confidence intervals)
☐	☒	For null hypothesis testing, the test statistic (e.g. *F*, *t*, *r*) with confidence intervals, effect sizes, degrees of freedom and *P* value noted<br>*Give P values as exact values whenever suitable.*
☒	☐	For Bayesian analysis, information on the choice of priors and Markov chain Monte Carlo settings
☒	☐	For hierarchical and complex designs, identification of the appropriate level for tests and full reporting of outcomes
☒	☐	Estimates of effect sizes (e.g. Cohen's *d*, Pearson's *r*), indicating how they were calculated

Our web collection on statistics for biologists contains articles on many of the points above.

Software and code

Policy information about availability of computer code

Data collection	no software was used for data collection
Data analysis	We used the following previously published software: EAGER (v 1.92.54); AdapterRemoval (v 2.2.0); BWA aln/samse programs (v 0.7.12); DeDup v0.12.2; pileupCaller (https://github.com/stschiff/sequenceTools); mapDamage (v 2.0); schmutzi; with ANGSD (v 0.910); EIGENSOFT package (v 6.0.1); ADMIXTURE (v 1.3.0); PLINK (v 1.90); ADMIXTOOLS package (v 4.1); Geneious (v11.0.4); the online tool "haplosearch"; the online tool "haplogrep"; Phylotree; yHaplo; Samtools mpileup (v 1.3); SwissCal 1.0

For manuscripts utilizing custom algorithms or software that are central to the research but not yet described in published literature, software must be made available to editors/reviewers. We strongly encourage code deposition in a community repository (e.g. GitHub). See the Nature Research guidelines for submitting code & software for further information.

Data

Policy information about availability of data

All manuscripts must include a data availability statement. This statement should provide the following information, where applicable:

- Accession codes, unique identifiers, or web links for publicly available datasets
- A list of figures that have associated raw data
- A description of any restrictions on data availability

All Alignment data (BAM) produced in this study is deposited in the European Nucleotide Archive (ENA) under the accession numbers (Study PRJEB24794). Other data supporting the findings of the study are available in this article and its Supplementary Information files, or from the corresponding authors upon request.

Field-specific reporting

Please select the one below that is the best fit for your research. If you are not sure, read the appropriate sections before making your selection.

☒ Life sciences ☐ Behavioural & social sciences ☐ Ecological, evolutionary & environmental sciences

For a reference copy of the document with all sections, see nature.com/documents/nr-reporting-summary-flat.pdf

Life sciences study design

All studies must disclose on these points even when the disclosure is negative.

Sample size	Sample sizes for ancient populations depended solely on the availability of archaeological samples and on ancient DNA preservation. A total of 10,000 markers covered per individual post-capture was considered as the lower threshold for he individual to be included in the analysis.
Data exclusions	Five samples were screened and found positive for DNA presence but excluded for analysis based on preset criteria: Two samples (ZHBD and ZHF) were excluded due to high contamination estimates (higher than 10% contaminating DNA) and samples ZHF, ZQI, ZHB, ZQM due to low post capture coverage (under 10,000 markers covered once) insufficient for quality control. An overview of the screened samples (including the ones not analyzed) is given in Supplementary Data 1.
Replication	Not applicable
Randomization	Not applicable
Blinding	Not applicable

Reporting for specific materials, systems and methods

We require information from authors about some types of materials, experimental systems and methods used in many studies. Here, indicate whether each material, system or method listed is relevant to your study. If you are not sure if a list item applies to your research, read the appropriate section before selecting a response.

Materials & experimental systems

n/a	Involved in the study	
☒	☐	Antibodies
☒	☐	Eukaryotic cell lines
☐	☒	Palaeontology
☒	☐	Animals and other organisms
☒	☐	Human research participants
☒	☐	Clinical data

Methods

n/a	Involved in the study	
☒	☐	ChIP-seq
☒	☐	Flow cytometry
☒	☐	MRI-based neuroimaging

Palaeontology

Specimen provenance	*Provide provenance information for specimens and describe permits that were obtained for the work (including the name of the issuing authority, the date of issue, and any identifying information).*
Specimen deposition	The extracts and DNA libraries prepared from the ancient specimens are housed in the laboratory facilities of the Max Planck Institute for the Science of Human History, Jena, Germany and are available upon request from the corresponding authors.
Dating methods	*If new dates are provided, describe how they were obtained (e.g. collection, storage, sample pretreatment and measurement), where they were obtained (i.e. lab name), the calibration program and the protocol for quality assurance OR state that no new dates are provided.*

☒ Tick this box to confirm that the raw and calibrated dates are available in the paper or in Supplementary Information.
