# Supplementary material for: Late Pleistocene human genome suggests a local origin for the first farmers of central Anatolia
Source: Nat Commun. 2019 Mar 19;10:1218. doi: 10.1038/s41467-019-09209-7 (PMC6425003; doi:10.1038/s41467-019-09209-7)
Supplement: Supplementary file 4 — Description of Additional Supplementary Files [file 41467_2019_9209_MOESM4_ESM.pdf]

Description of Additional Supplementary Files

Supplementary Data 1:

An overview of skeletal material screened for ancient DNA in this study. The total number of sequenced reads (Raw Reads), the proportion of endogenous human DNA and the levels of DNA damage in the mapped reads (given as the deamination level at the 5' terminal position of the mapped reads) are provided for both shotgun libraries and libraries enriched for mitochondrial DNA when available. Samples with above 0.2 % endogenous DNA and over 7 % DNA damage on 5' were defined as positive for nuclear ancient human DNA content (pos; highlighted in green) and samples with above 10 % endogenous DNA and over 7 % DNA damage on 5' (post-capture) were defined as positive for mitochondrial ancient human DNA content. Positive samples analyzed in this study are highlighted in green. Samples that are reported but not analyzed (based on either low post capture coverage or high contamination estimates) are highlighted in orange. The ones not analyzed due to high contamination estimates (> 10%) are further marked with an asterisk.

Supplementary Data 2:

An overview of the main analysis groups used in this study. Listed are published and new key populations used for analysis in this study. The average date is given as the average of the 95.4% date range in calBP (defined as 1950 CE).

Supplementary Data 3:

Differential affinity of ancient Anatolians tested by D(Anatolian pop1, Anatolian pop2; test, Mbuti). The investigated ancient Anatolian populations are given in "pop1" and "pop2" while global modern and ancient "test" populations are given in "pop 3", "Mbuti" is used as the outgroup. The Z scores were calculated from a 5 cM block jackknifing standard error.

Supplementary Data 4:

Symmetry testing of Levantine groups by D(KFH2/BAJ001, Levant_N/Natufian; test, Mbuti). Global modern and ancient "test" populations are given in "pop 3", "Mbuti" is used as the outgroup. The Z scores were calculated from a 5 cM block jackknifing standard error.

Supplementary Data 5:

Differential affinity of European hunter-gatherers and Near Eastern hunter-gatherers tested by D(European HG pop1, European HG pop2; Near Eastern HG, Mbuti/Altai Neanderthal). The Z scores were calculated from a 5 cM block jackknifing standard error. The tests were filtered for more than 30,000 overlapping SNPs (nSNPs) between the four tested populations.

Supplementary Data 6:

Mitochondrial haplotypes and haplogroup of the studied samples. Green indicates positions not covered, red positions covered with ≤5 reads and blue positions in which the reads harbouring the reported SNP were ≤5.

Supplementary Data 7:

Allele counts in SNP positions related to phenotypic traits. For each reference SNP (SNP ID) we provide the corresponding chromosome (Chr), the position on the Hg19 human reference (Position), the reference allele (Ref) and the alternative one (Alt), both on the positive strand, the gene name (Gene) and the alternative allele phenotype. The counts of sequenced reads covering each “Ref” and “Alt” allele are given for each of the reported individuals.

Supplementary Data 8:

Sequencing statistics of negative controls. For each negative control the mean coverage after target enrichment is given. The levels of DNA damage is given as the deamination level at the 5' and 3' terminal positions of the mapped reads.

Supplementary Data 9:

Pairwise mismatch rates between published and newly reported Boncuklu (AAF) individuals. The total number of overlapping SNP positions between the two paired individuals (nSNPs), the number of mismatching SNPs (nmismatch) and the proportion of overlapping SNPs out of the total number of mismatching SNPs (pmismatch) are given for each pair. Individual estimated to be first degree related are shown in bold.

Supplementary Data 10:

Symmetry test of all reported Boncuklu (AAF) individual pairs by D(Ind1, Ind2; test, Mbuti). Global modern and ancient “test” populations are given in “pop 3”, “Mbuti” is used as the outgroup. The Z scores were calculated from a 5 cM block jackknifing standard error.

Supplementary Data 11:

Permutation test of D(AAF, AHG; test, Mbuti). In each test, AAF* includes AHG and all AAF individuals except the one in Pop2. Global modern and ancient “test” populations are given in “pop 3”, “Mbuti” is used as the outgroup. The Z scores were calculated from a 5 cM block jackknifing standard error.
